# Low-cost machine learning prediction of excited state properties of iridium-centered phosphors†

**DOI:** 10.1039/d2sc06150c

**Published:** 2023-01-05

**Authors:** Gianmarco G. Terrones, Chenru Duan, Aditya Nandy, Heather J. Kulik

**Affiliations:** a Department of Chemical Engineering, Massachusetts Institute of Technology Cambridge MA 02139 USA; b Department of Chemistry, Massachusetts Institute of Technology Cambridge MA 02139 USA

## Abstract

Prediction of the excited state properties of photoactive iridium complexes challenges *ab initio* methods such as time-dependent density functional theory (TDDFT) both from the perspective of accuracy and of computational cost, complicating high-throughput virtual screening (HTVS). We instead leverage low-cost machine learning (ML) models and experimental data for 1380 iridium complexes to perform these prediction tasks. We find the best-performing and most transferable models to be those trained on electronic structure features from low-cost density functional tight binding calculations. Using artificial neural network (ANN) models, we predict the mean emission energy of phosphorescence, the excited state lifetime, and the emission spectral integral for iridium complexes with accuracy competitive with or superseding that of TDDFT. We conduct feature importance analysis to determine that high cyclometalating ligand ionization potential correlates to high mean emission energy, while high ancillary ligand ionization potential correlates to low lifetime and low spectral integral. As a demonstration of how our ML models can be used for HTVS and the acceleration of chemical discovery, we curate a set of novel hypothetical iridium complexes and use uncertainty-controlled predictions to identify promising ligands for the design of new phosphors while retaining confidence in the quality of the ANN predictions.

## Abstract 2

Neural networks are used to predict iridium phosphor excited state properties at accuracy competitive with TDDFT, enabling high-throughput screening.

## Graphical abstract

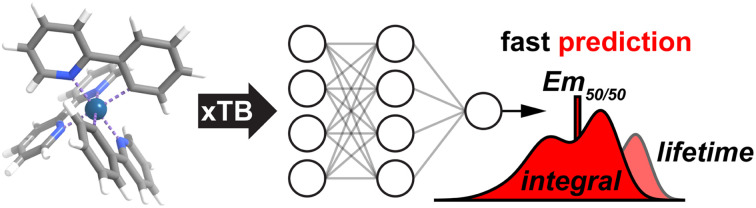

## 1. Introduction

Interactions between light and matter underpin phenomena ranging from photovoltaics^1^ to photosynthesis^2^ to bioluminescence,^3^ and the design of functional materials that can leverage these interactions has led to significant technological advancements.^4–6^ Exemplary of these advancements are photoactive iridium complexes that have been investigated extensively due to their applications in lighting and display technology,^7–10^ photocatalysis,^11–13^ and bioimaging.^14,15^ The spin–orbit coupling (SOC) characteristic of iridium causes these complexes to efficiently convert excitons into light or chemical energy.^16^ Simultaneously, iridium uniquely limits nonradiative decay rates by destabilizing a metal-centered (^3^MC) triplet excited state due to strong metal–ligand bonding,^17^ further improving efficiency. In iridium-centered complexes, the judicious selection of ligands allows for the modulation of phosphorescence color (*i.e.*, emission wavelength) and efficiency/brightness by modulating excited state lifetime and photoluminescence quantum yield.

The desired excited state properties in these highly tunable phosphors are application-dependent. In these complexes, emission energies span the visible spectrum, with complexes at the extremes of the distribution emitting red (1.6 eV)^18^ or blue light (2.8 eV).^19^ Furthermore, excited state lifetimes in these complexes are on the scale of microseconds, with shorter lifetimes (under 2 μs)^16^ preferred for displays and longer lifetimes preferred for photocatalysis^11^ and bioimaging.^14^ In addition, for display technologies and bioimaging a high photoluminescence quantum yield is desired. The accurate prediction of these excited state properties will enable the discovery of novel iridium complexes for vibrant display technologies and green photocatalysis.

To screen a large number of compounds, computational modeling with time-dependent density functional theory (TDDFT) can be used for affordable predictions of some properties of transition metal complexes. While TDDFT methods are commonly employed to estimate emission energies,^20–28^ the calculation of lifetimes and quantum yields is more challenging both from an accuracy and a computational cost perspective. The calculation of lifetime^23,29–33^ requires the inclusion of SOC in TDDFT to estimate the transition dipole moment between the sublevels of the excited triplet (*i.e.*, T_1_) and the ground state (*i.e.*, S_0_). The calculation of photoluminescence quantum yield further requires the calculation of nonradiative rates, which entails the use of thermal vibration correlation function rate theory^34–36^ and excited state geometry optimization.^36,37^ Thus, while *ab initio* computational methods have provided valuable insight into the properties of iridium complexes, they are computation-intensive, requiring around one day of computation time per complex for the least-demanding calculations, and may not reach the accuracy required to enable rational design.

Supervised machine learning (ML) has emerged as a powerful complement to *ab initio* methods in recent years due to its capacity to reproduce *ab initio* results at significantly lower cost,^38–42^ enabling the screening of vast regions of chemical space.^43^ Furthermore, ML models can be trained on experimental data, enabling the prediction of properties that challenge *ab initio* methods, such as material stability.^44^ With regard to excited state properties, ML models have been successfully applied for the prediction of phosphorescence energies,^45^ fluorescence rates,^46^ and fluorescence energies and quantum yields^47^ after training on *ab initio* or experimental data. While ML models were first demonstrated for accelerating DFT screening of Ir catalysts in 2020,^48^ the extension to directly predicting experimental catalytic^49^ or photophysical^50,51^ properties has only recently been demonstrated. The need to predict and optimize multiple properties of iridium complexes that challenge TDDFT motivates the continued extension of ML to the direct prediction of experimental properties.

In this work, we use ML to predict three key properties of iridium complexes: Em_50/50_ (mean emission energy), excited state lifetime, and emission spectral integral (brightness). We train and evaluate artificial neural networks (ANNs) on a recent experimental dataset^52^ of 1380 iridium(iii) phosphors and their properties. This large experimental dataset represents an ideal scenario for ML model training given its uniformity in comparison to acquiring heterogeneous data from multiple sources and conditions. We show that features generated with density functional tight binding lead to the most predictive ANN performance and generalization on out-of-sample data. Using these features, we identify trends in phosphor properties, and we extend our models to a new set of hypothetical iridium phosphors. These experimentally-informed ANNs enable fast, accurate prediction of iridium phosphor properties for the rapid exploration of chemical space when paired with uncertainty control to only apply the ANNs where they are likely to be predictive.

## 2. Data and representations

### 2.1. Dataset

**Fig. 1:**
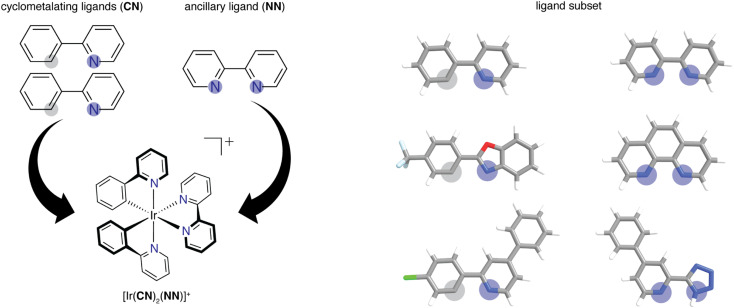
(Left) Schematic of how two identical CN ligands and one NN ligand comprise each of the iridium phosphors studied in this work. Coordinated nitrogen (carbon) atoms are indicated with blue (gray) circles. (Right) Examples of CN and NN ligands in the experimental dataset of 1380 iridium phosphors. Atoms are colored as follows: white for hydrogen, gray for carbon, blue for nitrogen, red for oxygen, light blue for fluorine, and green for chlorine.

We built the structures of bidentate ligands used in the experimental study of DiLuzio *et al.*^52^ on Ir(iii) complexes of the form [Ir(CN)_2_(NN)]^+^ (Fig. 1). We assigned each ligand as either cyclometalating (CN) or ancillary (NN), as determined by the two iridium-coordinating atom identities. We studied the same 60 CN ligands and 23 NN ligands from the prior experimental study,^52^ excluding only the monodentate DMSO ligand in the prior work, giving rise to a combinatorial set of 1380 [Ir(CN)_2_(NN)]^+^ phosphor complexes. This set of 83 ligands will be referred to as the high-throughput ligand set (HLS), and we use the same labeling as in the prior study when referring to individual ligands (ESI Tables S1 and S2†). We used experimental data from the prior study^52^ on the three target properties, Em_50/50_, excited state lifetime, and emission spectral integral. The experimental values for these properties were reported for each of the 1380 iridium phosphors in DMSO solvent and were used for ML model training and performance assessment (ESI Fig. S1†). CN ligands were generated in their neutral form (*i.e.*, with a proton added) for featurization. Because of this, all ligands are neutral with the exception of three NN ligands (ESI Text S1†). After ligand construction using the draw tool in Avogadro v1.1.2 (ref. 53 and 54) and force field (*i.e.*, UFF) optimization, we used these ligands to generate the structures of all possible iridium complexes with one distinct type of CN ligand and one NN ligand using molSimplify v1.6.0 (ref. 55 and 56) and force field optimized again.

### 2.2. Feature sets

When developing machine learning models, it is important to strike the right balance between interpretability (*i.e.*, through features that relate to physical properties) and generalizability (*i.e.*, a model that performs well on complexes for which it was not trained). Thus, we evaluated and compared eight representations of the iridium complexes to identify the most suitable set of features for training ML models to predict Ir phosphor properties. The feature sets can be categorized into those based on substructure/fingerprints (*i.e.*, Morgan, Dice), those based on graph descriptors (*i.e.*, whole-complex revised autocorrelations, referred to as RACs,^57^ ligand-only RACs, and Coulomb-decay RACs,^58^ referred to as CD-RACs), and those based on electronic structure calculations (*i.e.*, xTB, ωPBEh, and B3LYP). For the substructure feature sets, we generated Morgan fingerprints,^59,60^ which have been used previously in machine learning chemistry applications,^61–66^ by one-hot encoding of groups of atoms in a structure. We computed these with a radius of three and 2048 bits on the isolated CN and NN ligands to capture the presence and absence of chemical substructures. We also generated Dice similarity coefficients^59^ of ligand Morgan fingerprints. In this approach, we separately compare the Morgan fingerprints of the CN and NN ligand of each new iridium complex to all HLS CN or NN ligand Morgan fingerprints through the Dice similarity metric, which is a common measure to quantify the connectivity similarity of two molecules (ESI Text S2 and Table S3†). The Dice feature set size is determined by the number of training set HLS ligands (83 features in the random split, 78 in the grouped split, see next in Features for ANN models and performance). Dice similarity was selected after we found it outperforms the commonly employed Tanimoto similarity (ESI Table S4†).

Unlike similarity or fingerprint feature sets, graph-based representations capture the entire structure of the molecule, requiring any machine learning model trained on the graph representation to emphasize which components of the molecule matter most but with the potential benefit of generalizing to ligands that had not been seen before. For the graph-based feature sets, we generated RACs^57,67^ for both the isolated ligands and the full iridium complex structures (ESI Text S2†). RACs are connectivity-based representations that have shown good performance for transition metal complex (TMC) property prediction.^43,57,68^ For RACs, a TMC is represented as a molecular graph, with vertices for atoms and unweighted (*i.e.*, no bond length or order information) edges for bonds. Each RAC feature is the sum of products or the sum of differences of heuristic atomic properties at depth *d* on a TMC molecular graph, where *d* indicates the number of edges separating the starting and ending atoms (ESI Text S2†). The RACs include features that span the entire complex as well as weighted averages over the equatorial ligands and axial ligands, where CN and NN ligands may be classified as both when they are present in both the equatorial plane and axial position. We used the largest set of heuristic properties described in previous work, including both group number^69^ and number of bonds,^58^ leading to a final RAC feature set that contains 196 features (ESI Table S5†). For the ligand-only RAC feature set, we generated full-scope product RACs (*i.e.*, all atoms are used as starting atoms) on isolated CN and NN ligands for each TMC. We concatenated individual feature vectors for the CN ligands and NN ligands with equal weighting for each ligand type. The ligand-only RAC feature set contains significantly fewer (*i.e.*, only 70) total features than the RAC feature set (ESI Table S6†). We also generated Coulomb-decay RACs^70^ on the iridium complex structures that were optimized with UFF (see Dataset). CD-RACs are a variant of RACs that also encode distances between the atoms in the RAC feature (ESI Text S2†). The CD-RAC feature set contains Coulomb-decay versions of the features in the RAC feature set but is of higher dimension (*i.e.*, 222 features) due to the added information from the geometry (ESI Table S7†).

Finally, we computed descriptors obtained from electronic structure theory, which were selected because they can be expected to correlate directly to the photophysical properties of the Ir complexes. Specifically, we selected electronic properties of the isolated ligands due to the lower computational cost in comparison to whole-complex properties. These ligand-based descriptors include the highest occupied molecular orbital (HOMO) and lowest unoccupied molecular orbital (LUMO) energies of each ligand type, the ionization potential (IP) and electron affinity (EA) of each ligand type, and the partial charges (*i.e.*, Mulliken) of each of the metal-coordinating atoms (ESI Table S8†). For the xTB feature set, we utilized a specially reparametrized^71,72^ vertical ionization potential and electron affinity-focused version of GFN1-xTB, a low-cost, semi-empirical tight binding method that has parameters for most elements in the periodic table.^73^ We calculated ligand-only xTB features on UFF-optimized CN and NN ligands. The electronic structure features consist of quantum mechanical properties of the CN and NN ligands of a phosphor, and are in some cases correlated to each other (*e.g.*, the HOMO and the EA of a ligand are closely related, ESI Table S8 and Fig. S2†). For the B3LYP and ωPBEh DFT feature sets, we performed density functional theory (DFT) calculations on isolated CN and NN ligands using the B3LYP^74–76^ or ωPBEh^77^ exchange correlation functionals respectively (see Computational details). Mulliken charges were used after they were found to outperform natural bond orbital (NBO) charges (ESI Table S9†).

## 3. Results and discussion

### 3.1. Features for ANN models and performance

**Fig. 2:**
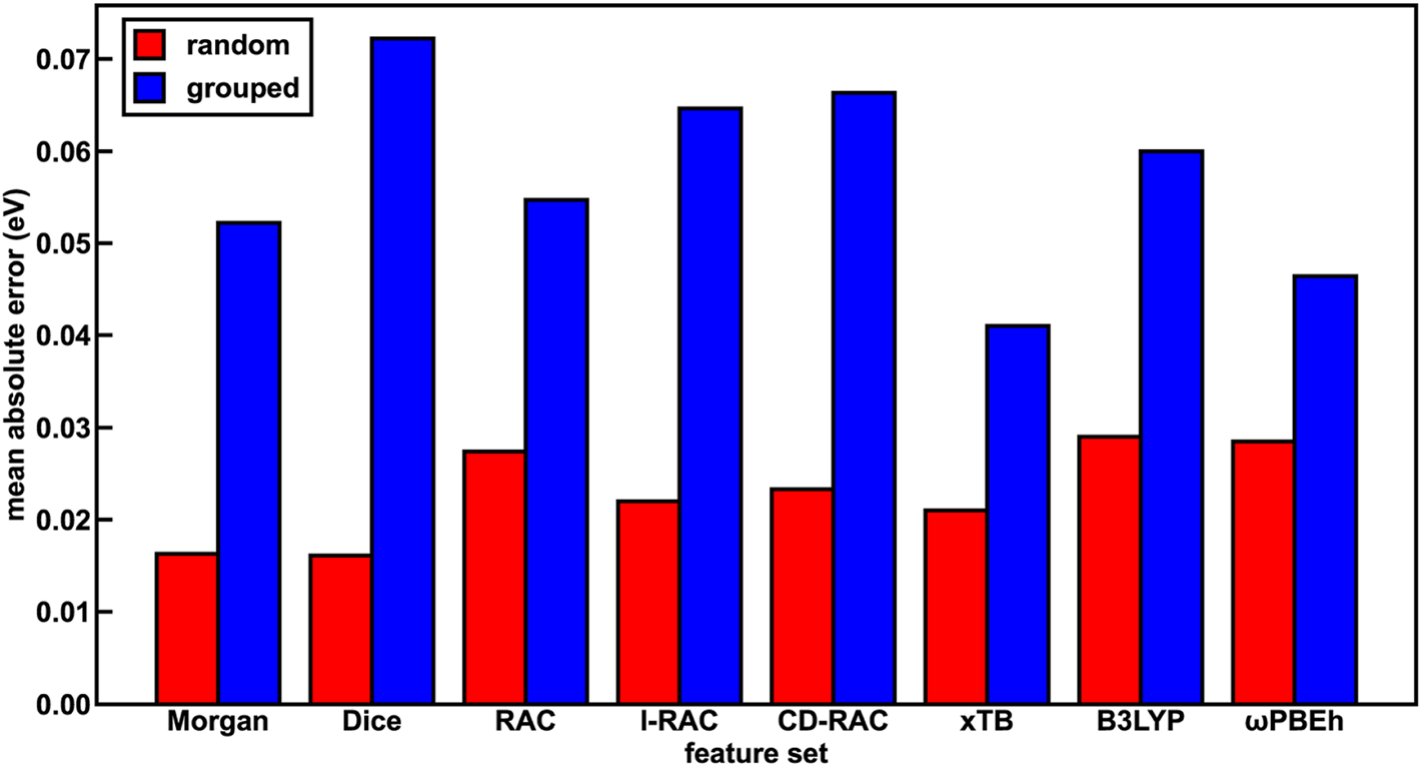
The test set performance of ANNs trained on different feature sets in predicting Em_50/50_ (MAE, in units of eV) for both random (red bars) and grouped splits (blue bars). Here, l-RAC refers to ligand-only RACs.

The representation for the phosphor is a crucial piece in determining whether a machine learning (*i.e.*, ANN) model is likely to predict experimental properties accurately and to generalize to unseen complexes. A model and feature set that perform well on one property may perform poorly on another. We thus trained a total of 24 ANN models with each of the eight feature sets and the three target properties (Em_50/50_, excited state lifetime, and emission spectral integral) and assessed their prediction performance on both a random split and grouped split of the training data. Here, random split refers to an 85/15 train/test partition, whereas in the grouped split five ligands are present only in the test set and are consequently unseen by the ANNs during training (see Computational details). The grouped split provides a more stringent test of how well our models generalize. For the random split train/test partition, the Dice, Morgan, and xTB feature sets lead to the lowest errors across all three target properties, suggesting they fit the data the best. The B3LYP DFT and RAC feature sets lead to the largest model test set errors, while the CD-RAC, ligand-only RAC, and ωPBEh DFT feature sets exhibit intermediate performance (Fig. 2 and ESI Fig. S3, S4 and Tables S10–S13†). The composition-based Dice and Morgan feature sets lead to the best predictions, judged on the basis of scaled MAEs of 0.03 to 0.05 for the three target properties (ESI Tables S11–S13†). There is a substantial difference in performance between feature sets: the percent difference in mean absolute error (MAE) on the test partition of the random split of the data between using the optimal feature set and the worst feature set for mean emission energy is 80%, and a similar performance erosion is observed for the other two properties (*i.e.*, 59% for spectral integral and 40% for phosphorescence lifetime), suggesting that it is important to select the best feature set to yield good performance.

We rationalize the relative performance of each feature set in the assessment on the random split of the training data by considering what aspects of ligands and complexes the different feature sets capture. We attribute the predictive power of the Dice feature set to the fact that phosphorescent properties are very ligand-directed, and in the random split of the training data, each ligand is represented in both the train and test sets. We attribute the predictive power of the Morgan feature set in the random split of the training data to the identification of substructures in ligands that affect phosphor excited state properties by tuning energy levels and ligand rigidity. Thus, features encoding ligand similarity to previously observed ligands and substructures present in ligands lead to the best performance on the random split. On the other end of the spectrum, the ANNs trained and assessed on a random split of the data using the RAC feature set (scaled MAE: 0.05 to 0.08) likely perform most poorly because they include significant metal-local information that does not vary across this set, because all complexes have an iridium center and an identical first coordination shell (ESI Tables S5 and S11–S13†). Thus, in datasets with a single metal center where only the ligands vary, standard similarity-based feature sets perform best in describing ligand variation. However, we still had to determine whether such feature sets are useful for discovery of novel complexes.

With regard to the ligand-only electronic structure feature sets, we surprisingly observe improved ANN performance on a random split of the training data with the xTB feature set relative to the two DFT feature sets. While xTB is expected to be faster than DFT for feature generation, electronic structure properties from xTB alone should not necessarily be more accurate. The Em_50/50_ xTB model error is 30% lower than that of the corresponding B3LYP DFT model (*i.e.*, 0.021 eV *vs.* 0.029 eV). Nevertheless, most (*i.e.*, eight of twelve) xTB features have high (>0.5) linear correlation with their B3LYP DFT and ωPBEh DFT counterparts, as determined by Pearson correlation coefficients (ESI Table S14†). Thus, the reparametrized GFN1-xTB method provides reliable electronic structure information from which our ANNs can generate accurate predictions. The reparametrized xTB method^71^ is fitted to IP/EA values calculated with PW6B95/def2-TZVPD,^78^ and it is possible that this functional and basis set combination achieve more accurate calculated properties than those generated in our DFT feature sets, leading to better ANN learning with the xTB feature set.

To assess the utility of ANN models trained on each of the feature sets for discovery of out-of-distribution complexes, we repeated the ANN training process on a grouped split, where five ligands are present only in the test set and are consequently unseen by the ANNs during training (see Computational details). We used the same grouped split across each property prediction task. We find that the test accuracy of the ANNs trained and tested on the grouped split of the data is worse than that of the corresponding ANNs trained and tested on a random split of the data for all features due to the presence of unseen ligands in the test set (ESI Tables S15–S17†). This worsened performance is most significant for the spectral integral and Em_50/50_ target properties. Overall, the change in MAE averaged over all feature sets is significantly worse for these two properties (157% or 164% worse on average for spectral integral and Em_50/50_, respectively) than for phosphorescence lifetime (28% worse, ESI Table S18†).

With the grouped split, the predictive power of the xTB feature set is improved relative to the other feature sets (Fig. 2 and ESI Fig. S3, S4†). For Em_50/50_ prediction, the xTB feature set improves from the third-best feature set to the best feature set as a result of its scaled MAE increasing less than the best performers assessed on a random split of the training data (*i.e.*, 0.04 to 0.078 for xTB *versus* Dice 0.031 to 0.138, ESI Tables S18 and S19†). In practice, this means that the Em_50/50_ xTB MAE doubles from 0.021 eV to 0.041 eV, while the Dice MAE nearly quadruples from 0.016 eV to 0.072 eV. We attribute this particularly worsened performance of the Dice feature set to the loss of information about variations in ligand chemistry because features describing similarity to held out ligands are no longer in the feature set for the grouped split (ESI Table S3†). The poor generalizability of the Dice feature set can also be attributed to the pseudo one-hot encoding of ligands *via* the similarity scores. The xTB features, in contrast, convey physical information that extrapolates beyond the ligands seen in the training data. We ultimately chose the xTB feature set for further analysis in evaluating hypothetical complexes because the xTB feature set has favorable performance on the grouped split for all three properties, indicating that the ANNs using the xTB feature set generalize well.

**Fig. 3:**
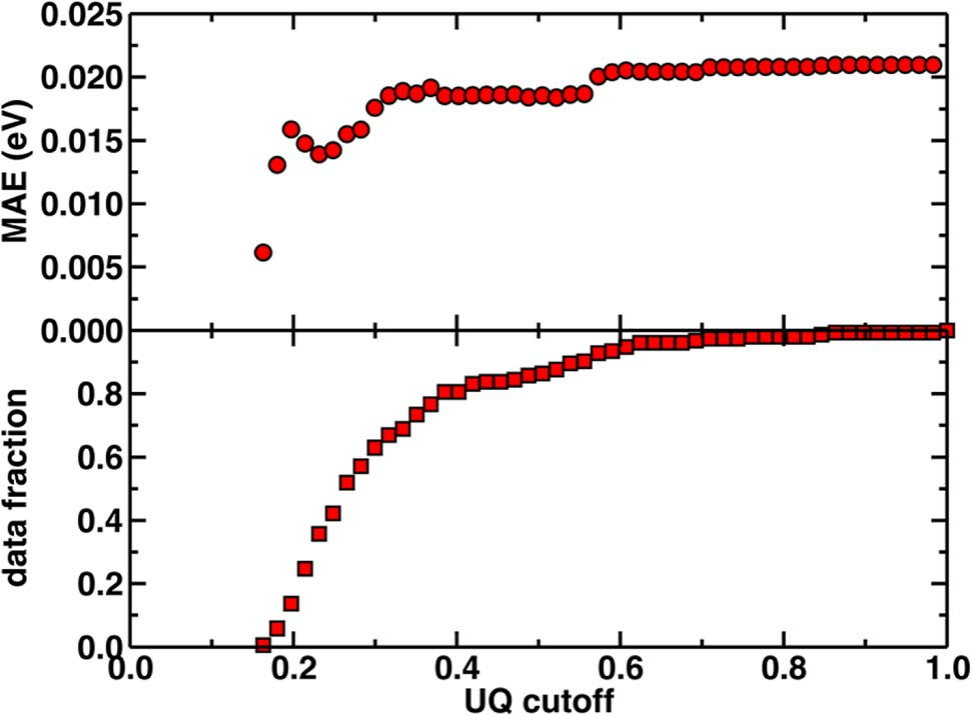
The uncertainty quantification (UQ) cutoff *versus* test set mean absolute error (in eV) of the ANN model trained on a random split of the training data with the xTB feature set for predicting Em_50/50_. The data fraction is the number of test set complexes under the corresponding UQ cutoff, and the MAE is calculated on this subset of complexes. The UQ metric used is the average latent space distance to the ten nearest neighbors in the training set following the protocol introduced in ref. 79. The UQ metric is normalized such that the largest UQ metric is scaled to 1.

Beyond test set error, one challenge for applying ML models to novel complexes is the need to know how confident we should be in their predictions (*i.e.*, to quantify the uncertainty). To quantify ANN uncertainty in predictions for new phosphors outside of our initial training set, we use the latent space distance as a measure of how similar a new phosphor is to the complexes used to train the model.^79^ The latent space is the last layer of an ANN, from which the final prediction is made *via* linear regression, and thus the distance in latent space of a new compound to training data should provide a representation of how different a new molecule is from training data according to the model. To confirm that this is a good measure of similarity that quantifies uncertainty for the current prediction task, we assessed the influence of latent space distance on test set prediction accuracy of the ANNs trained on a random split of the training data using xTB features as inputs. Following prior work,^79^ we computed the average distance to ten nearest neighbors in the latent space formed by the training set and discarded predictions on any test set phosphor with an uncertainty quantification (UQ) metric exceeding the cutoff. A nearly monotonic decrease in average model error *versus* UQ cutoff suggests the possibility to control the error of predictions on new phosphors by discarding any prediction with a large UQ metric (Fig. 3 and ESI Fig. S5, S6†). Based on analysis of this UQ metric, we choose to avoid making model predictions on novel complexes when the distance in latent space is significantly larger than that typically observed on the random split test set (*i.e.*, more than two standard deviations above the mean, see New compound exploration). Starting from a rescaled UQ metric where the most distant test complex is assigned a value of 1.0, the cutoff is largest for the spectral integral ANN (*i.e.*, 0.79) and somewhat smaller for the lifetime and Em_50/50_ ANNs (*i.e.*, 0.67 and 0.62).

### 3.2. Feature importance and trends

**Fig. 4:**
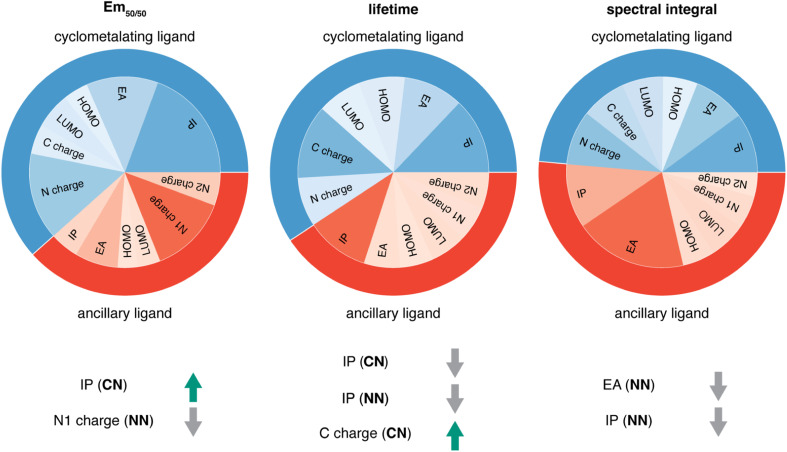
For each of the three target properties, the corresponding column indicates: (top) random forest feature importances of the xTB CN and NN features and (bottom) the correlation of the most important xTB features to the target property, where a green arrow indicates positive correlation and a gray arrow indicates negative correlation. For example, IP (CN) is positively correlated to Em_50/50_, while N1 charge (NN) is negatively correlated to Em_50/50_.

Given the high accuracy of the xTB-trained ANN models, we next sought to determine if simpler and more interpretable linear and random forest (*i.e.*, a series of binary decision trees) models trained on xTB features could attain similar accuracy. These models allow us to more transparently gain insight into which features most heavily influence phosphor property prediction. We trained random forest regression models that use xTB features to predict each of the three target properties. These random forest models have comparable performance to the ANNs and significantly outperform linear ridge regression models, (ESI Table S20 and Fig. S7†). Given the good performance of random forest models, we can analyze the most important features in these models to understand what features influence property prediction (*i.e.*, using impurity scores, Fig. 4). We find that xTB features of the CN ligand are more important than those of the NN ligand in predicting Em_50/50_ and lifetime. For both of these target properties, the sum of impurity-based importances of CN ligand features is approximately 50% larger than the corresponding sum for NN ligand features, consistent with the presence of two CN ligands for each NN ligand in the complexes. The large role of the CN ligand in determining Em_50/50_ can be explained by the partial localization of the phosphor complex HOMO on the CN ligand.^52^ In contrast, xTB features of the CN and NN ligand are equally important in predicting the spectral integral. This indicates that when tuning Em_50/50_ and lifetime, emphasis should be placed on selecting the CN ligand, whereas equal weight should be placed on varying CN and NN ligands to modify the spectral integral.

**Fig. 5:**
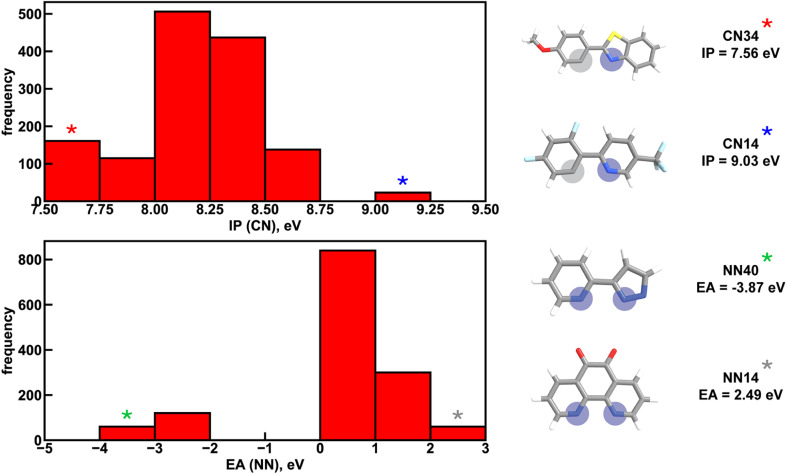
Distribution of two xTB features across the experimental dataset of 1380 iridium phosphors. IP (CN) refers to the ionization potential of the CN ligand and EA (NN) refers to the electron affinity of the NN ligand. Asterisks correspond to ligands at the extreme ends of the distributions, shown on the right. Coordinating nitrogen (carbon) atoms are indicated with blue (gray) circles. Atoms are colored as follows: white for hydrogen, gray for carbon, blue for nitrogen, red for oxygen, light blue for fluorine, and yellow for sulfur.

**Fig. 6:**
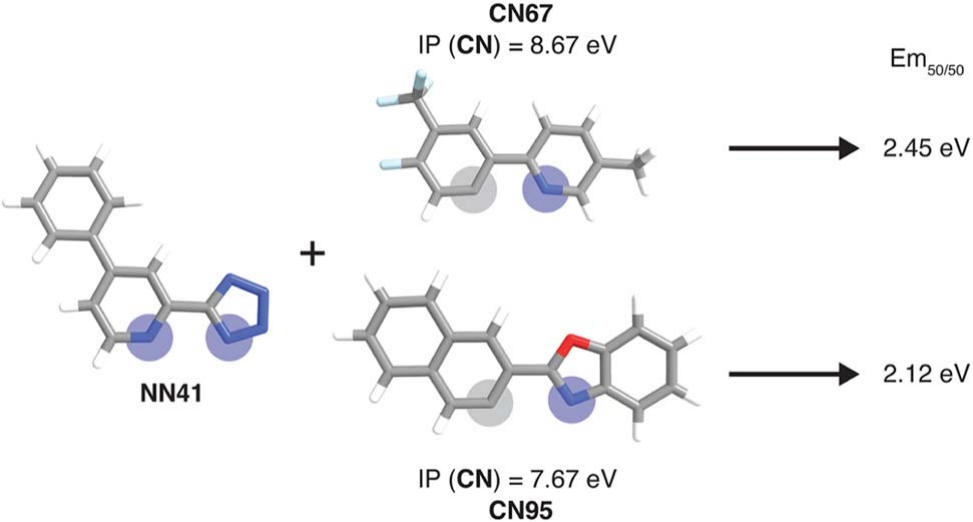
Example of a pair of complexes where the substitution of the CN ligand leads to a large Em_50/50_ property change. Coordinated nitrogen and carbon atoms are indicated with blue and gray circles, respectively. The relevant xTB features for the substituted ligands are shown. Atoms are colored as follows: white for hydrogen, gray for carbon, blue for nitrogen, red for oxygen, and light blue for fluorine.

Focusing more on Em_50/50_, we find that IP and EA are important for model predictions, as are the charges of metal-coordinating atoms (Fig. 4). Specifically, the top three xTB features for predicting Em_50/50_ are the IP of the CN ligand and two of the coordinating nitrogen charges (*i.e.*, N charge (CN) and N1 charge (NN)). The importance of IP (CN) conforms to prior observations that ligand energy levels affect emission energy.^80,81^ We also emphasize that these three xTB features vary significantly over the experimental dataset. The IP (CN) varies by nearly 1.5 eV (*i.e.*, from 7.56 eV to 9.03 eV), and the partial charges have a 0.1 a.u. range (*i.e.*, N charge (CN) from −0.35 a.u. to −0.24 a.u. and N1 charge (NN) from −0.37 a.u. to −0.28 a.u) (Fig. 5, 6 and ESI Fig. S8†). Thus, tuning these three features in a coordinated fashion should enable tuning of Ir phosphor complex Em_50/50_.

As was observed from our global analysis, the most important xTB features are different for lifetime and spectral integral predictions (Fig. 4). For predicting lifetime, IP features from CN and NN ligands dominate, and the most important charge feature is the C charge of the CN ligand. For spectral integral, the top three features are EA (NN), IP (NN), and IP (CN), none of which are obtained from charges. The different feature importances for different target properties suggest some possibility of orthogonal design, wherein one phosphor property is tuned independently of the others. Nevertheless, given that ionization potential and electron affinity of the CN and NN ligands play a large role for all three target properties, altering coordinating atom charge without significantly altering the IP/EA is likely the most direct way to target changes in Em_50/50_ or lifetime without altering the spectral integral.

Considering the most important xTB features as determined by random forest analysis, we further identified specific compounds with extreme (*i.e.*, high or low) experimental properties and compared how their xTB-computed features differed. For Em_50/50_, high emission energy complexes typically have a high IP (CN), while low emission energy complexes typically have a low IP (CN). The N1 charge (NN) tends to be more positive for low emission energy complexes than for high emission energy ones. However, it is more challenging to identify which features are most important for long lifetime. In general, complexes with long lifetimes have a lower IP for both CN and NN ligands combined with a higher C charge (CN), but there are numerous exceptions. In the case of spectral integral, EA (NN) and IP (NN) are lower for bright complexes with high spectral integrals.

To further identify specific examples of phosphors in the original experimental dataset that demonstrate the trends, we examined pairs of iridium complexes that differ only in the identity of one type of ligand. One such pair is [Ir(CN67)_2_(NN41)]^0^ and [Ir(CN95)_2_(NN41)]^0^ (Fig. 6). The former complex has an IP (CN) of 8.67 eV due to the electron-withdrawing fluorine groups on the cyclometalating ligand, while the latter complex has an IP (CN) of 7.67 eV. These values are on opposite ends of the IP (CN) distribution and contribute to Em_50/50_ values on opposite ends of the Em_50/50_ distribution, 2.45 eV and 2.12 eV, respectively (Fig. 5 and ESI Fig. S1†). The remaining five CN features for these two phosphors do not differ greatly from one another, underscoring the overriding effect of IP (CN). Similarly, increasing EA (CN) and EA (NN) can have a large effect on lifetime and spectral integral respectively (ESI Fig. S9 and S10†). These examples illustrate how differences in xTB features caused by ligand substitution correlate to shifts in phosphor properties.

**Fig. 7:**
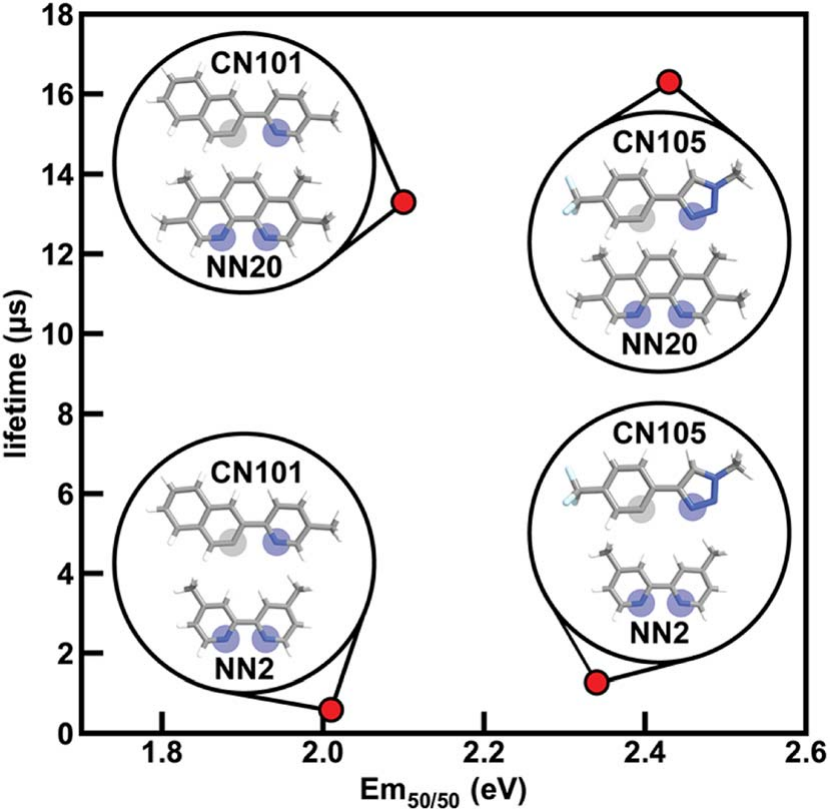
Four iridium phosphor complexes and the effect of substituting the CN or NN ligand on Em_50/50_ and lifetime indicated in the plot with structures shown as insets. Coordinated nitrogen (carbon) atoms are indicated with blue (gray) circles. Atoms are colored as follows: white for hydrogen, gray for carbon, blue for nitrogen, and light blue for fluorine.

To determine how ligand selection can allow for independent tuning, we consider the four complexes [Ir(CN101)_2_(NN2)]^+^, [Ir(CN101)_2_(NN20)]^+^, [Ir(CN105)_2_(NN2)]^+^, and [Ir(CN105)_2_(NN20)]^+^ that each differ by a single ligand. Changing the cyclometalating ligand from CN101 to CN105 leads to an increase in Em_50/50_ while having a small effect on phosphorescence lifetime, while changing the ancillary ligand from NN2 to NN20 leads to an increase in phosphorescence lifetime while having a small effect on Em_50/50_ (Fig. 7). The increase in Em_50/50_ when swapping CN101 for CN105 and the increase in lifetime when swapping NN2 for NN20 follows our observed trends of IP (CN) correlating positively to Em_50/50_ and IP (NN) correlating negatively to lifetime. Furthermore, the small change in Em_50/50_ when changing from NN2 to NN20 can be rationalized by the similar N1 charge (NN) between the two ancillary ligands. This example demonstrates how phosphor properties can be tuned orthogonally as guided by xTB features.

### 3.3. New compound exploration

We next aimed to demonstrate the utility of our ANNs in evaluating hypothetical complexes with ligands that were not in the training data but for which our models could make confident predictions. We applied one ANN for each property trained on a random split of the training data and used xTB features as inputs to screen hypothetical iridium complexes generated from CSD ligands (see Computational details and ESI Text S1†). Because the ANNs show better performance on random splits than grouped splits, they may be overfit to ligand chemistry present in the training data. Thus, we only considered hypothetical complexes under a UQ cutoff (*i.e.*, the distance in latent space) for all three ANNs. From a CSD screen, we identified 153 unique non-HLS CN ligands and 269 unique non-HLS NN ligands. Combining these new ligands with the HLS set led to 60 816 hypothetical complexes with at least one non-HLS ligand, of which 3598 hypothetical complexes fall within the UQ cutoff. This corresponds to inclusion of 70 unique non-HLS CN ligands and 42 unique non-HLS NN ligands in combination with each other or with HLS CN and NN ligands.

**Fig. 8:**
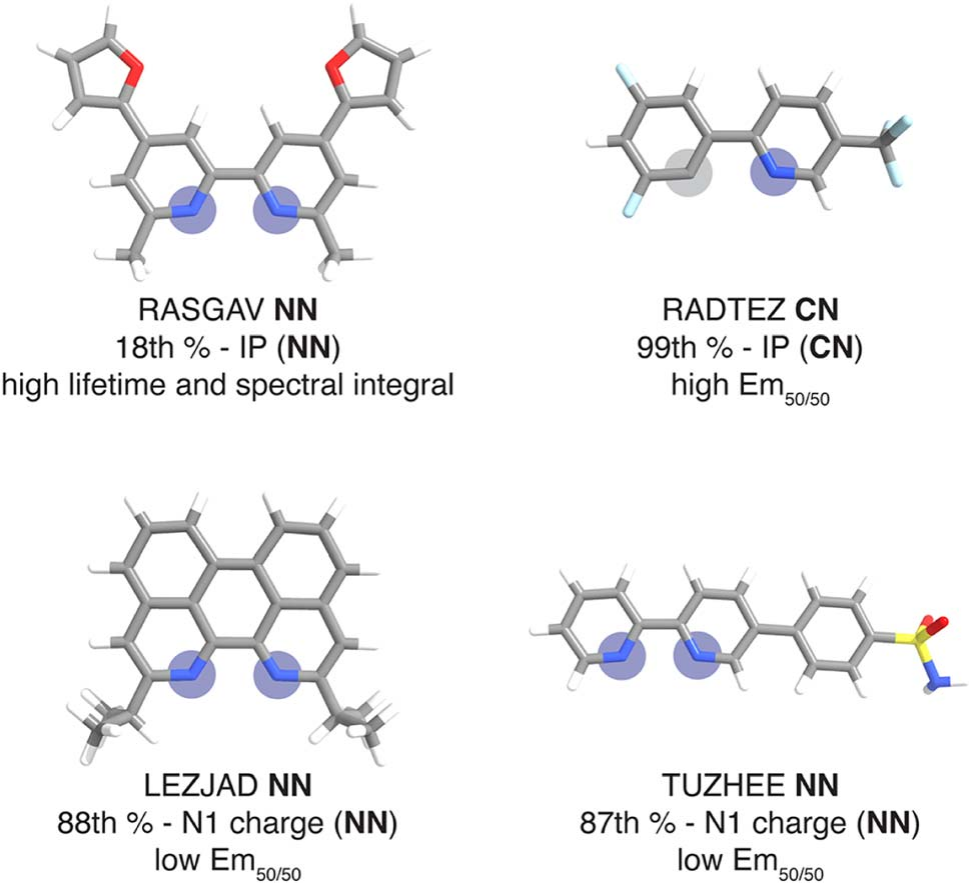
Ligands mined from the CSD that lead to very high or very low phosphor properties predicted by the ANNs along with their percentile rank of the relevant property in the context of the experimental complexes. Coordinated nitrogen and carbon atoms are indicated with blue and gray circles respectively. Atoms are colored as follows: white for hydrogen, gray for carbon, blue for nitrogen, red for oxygen, light blue for fluorine, and yellow for sulfur.

For this set of curated hypothetical complexes, we evaluated which ligands are present in the complexes with the highest and lowest ANN-predicted properties (ESI Fig. S11†). We find that specific ancillary ligands tend to be well-represented in complexes with extreme properties, indicating that phosphor properties are tuned by these ancillary ligands (ESI Table S21†). For example, the ligand that appears most often in hypothetical complexes with high predicted lifetime is the ancillary ligand from the CSD structure with refcode RASGAV. This conjugated ligand has a relatively low IP (NN) of 7.79 eV, which contributes to a longer lifetime following the previously identified trend (Fig. 4 and 8). Indeed, the other ancillary ligands that are well-represented in hypothetical complexes with extreme predicted lifetimes (NN ligands from complexes with refcodes FEQSEB, MIMYEO, TOTPAW, OVALEE, and MAXWIS) also follow the trend of low IP (NN) correlating to long lifetime (ESI Table S21†). We also note clear xTB feature trends in predictions for spectral integral and Em_50/50_. The low IP (NN) of the ancillary ligand from RASGAV leads to a hypothetical complex with one of the highest predicted spectral integrals (Fig. 4, 8 and ESI Table S22†). With regard to Em_50/50_, the fluorinated cyclometalating ligand from the CSD structure with refcode RADTEZ has a high ionization potential (9.24 eV). The high IP (CN) feature appears to contribute to a high emission energy, as the RADTEZ CN ligand is present in the three hypothetical complexes predicted to have the highest Em_50/50_ values (Fig. 4, 8 and ESI Table S22†). On the other hand, the ancillary ligands LEZJAD NN and TUZHEE NN have high N1 charge (NN) features, leading to their presence in the three hypothetical complexes with the lowest predicted Em_50/50_ values (Fig. 4, 8 and ESI Table S22†). Thus, we find that many ligands that lead to extreme hypothetical phosphor predicted properties follow our identified xTB feature trends from the experimental data. This lends interpretability to our model predictions and indicates that these predictions are derived from the electronic structure properties of the ligands.

**Fig. 9:**
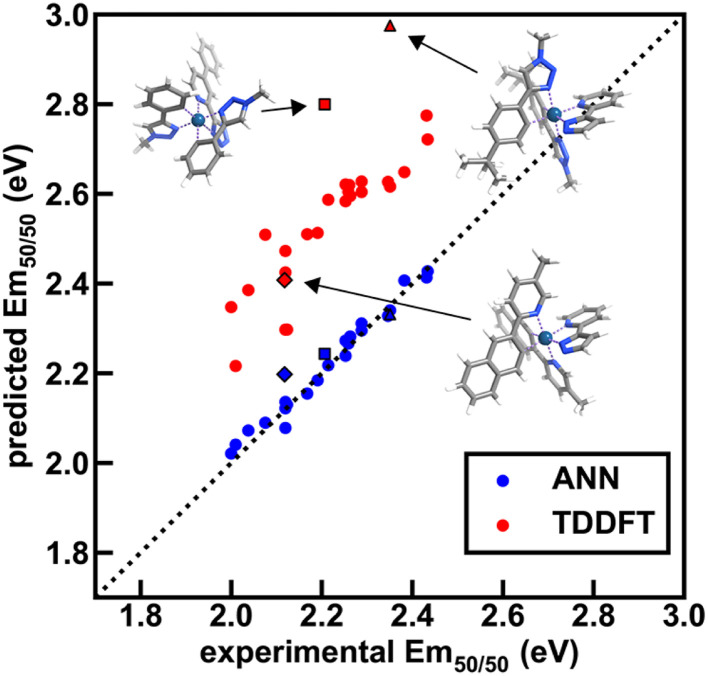
Comparison of ANN and TDDFT Em_50/50_ predictions to experiment (in eV) across 26 test set iridium complexes from the experimental dataset. These complexes were chosen to span the range of emission energies and lifetimes of the full set. TDDFT was carried out on optimized S_0_ singlet geometries using the B3LYP functional, and the energies of the three lowest triplet sublevels were averaged to approximate Em_50/50_; this approximation likely contributes to the worse performance of TDDFT relative to the ANN. Three high-error complexes ([Ir(CN101)_2_(NN40)]^0^, [Ir(CN107)_2_(NN41)]^0^, and [Ir(CN109)_2_(NN40)]^0^) are shown as insets, and their predicted and experimental Em_50/50_ values are shown with black borders and unique shapes (diamond, square, and triangle, respectively). In the insets, atoms are colored as follows: white for hydrogen, gray for carbon, blue for nitrogen, and dark blue for iridium. The dotted line is included as a reference and corresponds to perfect agreement between prediction and experiment.

To further validate performance of ANN models trained on a random split of the training data, we obtained TDDFT excited state energy and lifetime predictions and compared them to ANN predictions over complexes in both the experimental dataset and the uncertainty-controlled hypothetical dataset. Over a group of 26 representative test set complexes from the experimental dataset, we find that TDDFT overestimates the experimental emission energy by 0.3 eV on average, and further find that TDDFT predictions correlate with experiment less well than the Em_50/50_ ANN predictions (Fig. 9 and ESI Tables S23–S25†). These results show that the Em_50/50_ ANN achieves excellent performance. Even after applying a rigid downward shift to TDDFT energy predictions, they exhibit a larger spread around the experimental values than the predictions of our Em_50/50_ ANN. Over the same 26 complexes, TDDFT lifetime predictions trend with experiment and ANN predictions; however, unlike the case of Em_50/50_, TDDFT predictions outperform our lifetime ANN for complexes with long lifetimes (ESI Fig. S12†). This shortcoming of the lifetime ANN can be rationalized by the lower number of phosphors with long lifetimes in the experimental dataset used for model training (ESI Fig. S1 and Table S26†). Although we do not have ground-truth experimental data for our hypothetical set, we can still use TDDFT predictions to validate our ANN models. Over 21 representative hypothetical complexes, TDDFT energy predictions are on average 0.14 eV above Em_50/50_ ANN predictions, and TDDFT lifetime predictions trend with lifetime ANN predictions (ESI Fig. S13–S15 and Tables S27, S28†). These results indicate that uncertainty-controlled ANN predictions over the hypothetical set of complexes are reliable, although they may underestimate the lifetime of phosphors with long lifetimes. Thus, as long as they are paired with suitable UQ metrics, the ANNs are trustworthy tools for the identification of hypothetical complexes with desired excited state properties.

## 4. Conclusions

While *ab initio* methods like TDDFT are useful tools for studying excited states of iridium phosphors, they are computation-intensive and can also have insufficient accuracy, motivating the use of machine learning to leverage existing experimental data. Using experimental data on 1380 iridium phosphors, we trained ANNs to predict three experimental properties: Em_50/50_, excited state lifetime, and emission spectral integral. We found that features calculated with xTB led to the best overall performance across the three properties on out-of-sample complexes, outperforming the standard Morgan fingerprint features and features based on RACs. We then used random forest regression models to determine which xTB features most influence phosphor properties and found that high cyclometalating ligand ionization potential is indicative of high Em_50/50_, while high ancillary ligand ionization potential correlates to low lifetime and low spectral integral. These observations illustrate how phosphor properties can be altered through judicious ligand selection.

We next demonstrated how our ANNs can be applied to uncertainty-controlled chemical exploration by considering hypothetical iridium phosphors derived from ligands found in the CSD. We identified cyclometalating and ancillary ligands that lead to edge-of-distribution properties, such as an ancillary ligand predicted to result in both long-lifetime and high spectral integral phosphors. We confirmed the validity of these predictions by comparing to TDDFT, showing that for Em_50/50_ the ANN significantly outperforms TDDFT, while for lifetime the corresponding ANN performs well only in regimes of sufficient training data. To improve the lifetime model predictions for long-lifetime complexes, further engineering of the features (*e.g.*, to incorporate non-local properties such as ligand flexibility) could improve performance. The ANN models for iridium phosphor property prediction that we present here are promising tools for chemical screening and the acceleration of chemical discovery, as they can be used to quickly evaluate thousands of hypothetical iridium phosphors to identify promising candidates for follow-up synthesis.

## 5. Computational details

### 5.1. Feature and structure generation

We generated feature sets to represent the 1380 Ir phosphor complexes as inputs to ML models (see Feature sets). We generated all ligands using the draw tool in Avogadro v1.1.2 (ref. 53 and 54) and subsequently optimized them with UFF.^82^ We used molSimplify v1.6.0 for the generation of RAC feature sets on either ligands or complexes.^55,56^ The xTB features were generated using xTB 6.4.0,^73^ and DFT-based features were generated using the B3LYP^74–76^ or ωPBEh^77^ functional with the LACVP* basis set implemented in the TeraChem v1.9-2018.11-dev^83,84^ program. For the generation of the Morgan and Dice feature sets, we used RDKit 2021.9.2 (ref. 85) both for Morgan fingerprints and the Dice similarity coefficients evaluated on Morgan fingerprints.

For DFT electronic structure descriptors, ligand geometries were geometry optimized with neutral charge and singlet spin multiplicity using DFT in TeraChem. Geometry optimizations used the L-BFGS algorithm in translation rotation internal coordinates (TRIC)^86^ as implemented in TeraChem to the default tolerances of 4.5 × 10^−4^ hartree per bohr for the maximum gradient and 1 × 10^−6^ hartree for the change in energy between steps. We then performed single-point energy calculations on the optimized neutral ligand geometries at two different charges: +1 and −1 (ESI Text S1†). We used the LACVP* basis set, which for the HLS ligands corresponds to the LANL2DZ^87^ effective core potential for Br and the 6-31G* basis set for all remaining elements. We calculated all non-singlet states with an unrestricted formalism and singlet states with a restricted formalism. Level shifting of 0.25 Ha was employed on both virtual and occupied orbitals to facilitate self-consistent field convergence. We used the hybrid DIIS^88^/A-DIIS^89^ scheme for the self-consistent field procedure. We used TeraChem dynamic precision and a grid with approximately 3000 points per atom. Like the xTB feature set, DFT-generated features encode electronic structure information (ESI Table S8†).

### 5.2. ML models

We trained multiple artificial neural networks (ANNs) with each of the eight feature sets to predict three target properties: Em_50/50_, excited state lifetime, and emission spectral integral. For all ANNs trained on a random split of the data, we used a random 70%/15%/15% train/validation/test split of the 1380 complexes from the prior study.^52^ We find that results are robust to a random split with a larger test set allocation (56%/14%/30%, ESI Table S29†). To assess the generalizability of the ANNs, we also carried out grouped splits where we excluded from the training and validation data any complex containing a ligand from a select subset of CN and NN ligands. For the excluded ligands, we selected CN21, CN103, CN104, NN20, and NN43 after determining these to be the most dissimilar HLS ligands relative to the other HLS ligands as measured through Dice similarities of Morgan fingerprints (ESI Tables S1, S2 and S30†). If one of the 1380 complexes contains one or more of these ligands, it is held out from the training set. We pre-processed features by normalizing each feature to a zero mean and unit variance over the train and validation data and removed any invariant features (ESI Text S2†). For ANNs predicting lifetime and Em_50/50_, we excluded 356 complexes with low luminescent intensity (*i.e.* spectral integral less than 1 × 10^5^ photon counts) from ANN training and performance evaluation due to the greater noise in lifetime and Em_50/50_ measurements for dim Ir phosphors.

We built ANNs with Keras 2.4.3 with TensorFlow 2.3.0 as the backend.^90,91^ Both bypass and residual layers were included as possible components of the ANN architecture for selection during hyperparameter optimization. Hyperparameters for each ANN were chosen using Hyperopt^92^ with 200 evaluations, as judged by the mean absolute error of the model on the validation data. The built-in tree of Parzen estimator^93^ algorithm in Hyperopt was used to select model hyperparameters. We used these chosen hyperparameters to train the final model on the combined train and validation data and evaluated performance on the test set (ESI Table S31†). All ANN models were trained with the AMSGrad variant^94^ of the Adam optimizer^95^ up to 2000 epochs. Dropout,^96^ batch normalization,^97^ and early stopping^98^ were applied to avoid over-fitting. The patience for early stopping was 100. We enforced a floor of zero for all predictions since negative predictions for Em_50/50_, lifetime, or spectral integral are unphysical. All machine learning models have been deposited online in a Zenodo repository.^99^

### 5.3. Out-of-distribution complexes

We identified hypothetical out-of-distribution iridium complexes which we enumerated combinatorially using CN and NN ligands not in the HLS. We selected these ligands by screening the CSD v5.42 + 2 updates, released in November 2020, for iridium complexes with two CN ligands and one NN ligand by specifying the first coordination sphere around iridium in a ConQuest 2021.1.0 search. Complexes selected by the screening were then examined by hand, and those that were not fit for analysis were eliminated (ESI Table S32†). The molSimplify code was used to identify unique ligands from the remaining complexes on the basis of their atom-weighted molecular graph determinants,^100^ and we used any ligands not already in the HLS in combination with the HLS to generate new hypothetical [Ir(CN)_2_(NN)]^+^ complexes (ESI Text S1†).

### 5.4. TDDFT calculations

For *ab initio* validation of predictions using TDDFT, iridium phosphors were first geometry optimized, and TDDFT was then run on the optimized geometries using the ORCA 5.0.1 (ref. 101) program. All calculations employed a C-PCM solvation correction^102^ to mimic DMSO. Singlet (*i.e.*, S_0_) geometry optimization was carried out using the B3LYP^74–76^ functional and the def2-TZVP^103^ basis set with D4 dispersion correction^104^ on structures generated by molSimplify. We found that using ground state singlet geometries instead of T_1_ triplet geometries as inputs to TDDFT leads to better agreement with experiment, although geometries do not differ greatly in their RMSD (ESI Table S25 and Fig. S16†). Emission energies calculated with B3LYP were found to correlate with experiment better than those calculated with the range-separated hybrid functionals CAM-B3LYP and ωB97X-D3BJ, motivating our use of B3LYP for TDDFT (ESI Fig. S17 and S18†). For TDDFT, the Zero-Order Regular Approximation (ZORA)^105^ was used. The SARC-ZORA-TZVP^106^ basis set was used for iridium and the ZORA-def2-TZVP basis set was used for all other elements along with the SARC/J auxiliary basis set. The TDDFT calculation included 25 roots. Quasi-degenerate perturbation theory spin–orbit coupling^107^ was enabled, and the Tamm–Dancoff approximation was disabled.

Due to relativistic SOC caused by iridium, the T_1_ manifold is split into three sublevels (zero-field splitting). For the *ab initio* calculation of emission energy, the energies of these three lowest triplet sublevels from the TDDFT calculation were averaged for each complex. For *ab initio* lifetime, radiative rate and radiative lifetime were calculated as in prior work^29–31,33,108^ using output from TDDFT calculations. The radiative rate *k*_*i*_ from a triplet sublevel *i* is given by:1
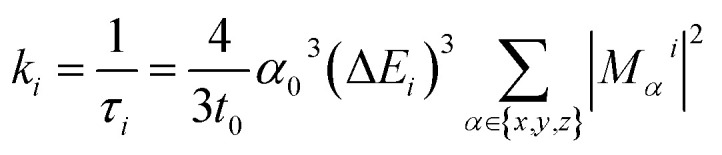
where *τ*_*i*_ is the radiative lifetime of sublevel *i*, *t*_0_ = (4π*ε*_0_)^2^ħ^3^/*m*_e_*e*^4^, *α*_0_ is the fine structure constant, Δ*E*_*i*_ is the excitation energy in atomic units from the ground state to the sublevel *i*, and *M*_*α*_^*i*^ is the *α*-axis projection of the transition dipole moment in atomic units between the ground state and the sublevel *i*.

The overall radiative lifetime from the three triplet sublevels is calculated as a Boltzmann average of radiative rates that depends on the energy differences between triplet sublevels.2
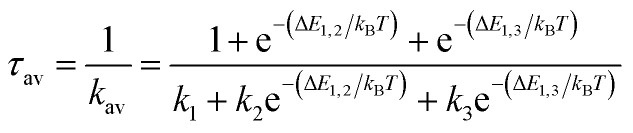
Δ*E*_1,2_ is the energy difference between sublevels 1 and 2, and Δ*E*_1,3_ is the energy difference between sublevels 1 and 3. *T* = 300 K was used. This equation for lifetime does not take into account nonradiative decay, which can be significant in some cases. In order to account for the DMSO solvent, the calculated radiative lifetime was divided by the square of the refractive index of DMSO according to the Strickler–Berg relationship^109^ in order to determine the final TDDFT lifetime.

## Data availability

The datasets supporting this article have been uploaded as part of the ESI.† The ANN models associated with this work are deposited on Zenodo and have the following permanent DOI: https://doi.org/10.5281/zenodo.7090416.

## Author contributions

Gianmarco G. Terrones: data curation, ML training, conceptualization, writing – original draft preparation, visualization; Chenru Duan: ML training, writing – reviewing and editing; Aditya Nandy: data curation, writing – reviewing and editing; Heather J. Kulik: writing – reviewing and editing, supervision, conceptualization.

## Conflicts of interest

The authors declare no competing financial interest.

## Supplementary Material

SC-014-D2SC06150C-s001

SC-014-D2SC06150C-s002
