# Supplementary material for: Low-cost machine learning prediction of excited state properties of iridium-centered phosphors
Source: Chem Sci. 2023 Jan 5;14(6):1419–33. doi: 10.1039/d2sc06150c (PMC9906783; doi:10.1039/d2sc06150c)
Supplement: SC-014-D2SC06150C-s002 [file SC-014-D2SC06150C-s002.pdf]

Electronic Supplementary Material (ESI) for Chemical Science.
This journal is © The Royal Society of Chemistry 2023

Electronic Supplementary Information for
Low-cost machine learning prediction of excited state properties of iridium-centered phosphors

Gianmarco G. Terrones[1], Chenru Duan[1,2], Aditya Nandy[1,2], and Heather J. Kulik[1,2]
[1]*Department of Chemical Engineering, Massachusetts Institute of Technology, Cambridge, MA 02139*
[2]*Department of Chemistry, Massachusetts Institute of Technology, Cambridge, MA 02139*

Contents

Table S1 Cyclometalating ligand structures and labels Page S3
Table S2 Ancillary ligand structures and labels Page S7
Figure S1 Histograms of the three target properties Page S9
Text S1 Electronic structure method and CSD details Page S10
Text S2 Feature set details Page S12
Table S3 Features in the Dice feature set Page S14
Table S4 Performance of different similarity features Page S14
Table S5 Features in the eRAC feature set Page S15
Table S6 Features in the ligand-only eRAC feature set Page S16
Table S7 Features in the CD-RAC feature set Page S17
Table S8 Features in the electronic structure feature sets Page S18
Figure S2 Correlation between xTB features Page S19
Table S9 Performance of different charge features Page S20
Figure S3 Feature set performance on lifetime Page S20
Figure S4 Feature set performance on spectral integral Page S21
Table S10 Random split feature set ranking Page S21
Table S11 Random split $Em_{50/50}$ performance Page S22
Table S12 Random split lifetime performance Page S22
Table S13 Random split spectral integral performance Page S22
Table S14 Correlation between xTB and DFT features Page S23
Table S15 Grouped split $Em_{50/50}$ performance Page S24
Table S16 Grouped split lifetime performance Page S24
Table S17 Grouped split spectral integral performance Page S24
Table S18 Random to grouped split performance difference Page S25
Table S19 Grouped split feature set ranking Page S25
Figure S5 Uncertainty quantification for the lifetime xTB ANN Page S26
Figure S6 Uncertainty quantification for the spectral integral xTB ANN Page S27
Table S20 Comparison of different ML models Page S27
Figure S7 Correlation between xTB features and target properties Page S28
Figure S8 Histograms of xTB features Page S29
Figure S9 Ligand substitution effect on lifetime Page S30
Figure S10 Ligand substitution effect on spectral integral Page S30
Figure S11 Sixteen CSD ligands that lead to extreme hypothetical complexes Page S31
Table S21 Well represented ligands in extreme hypothetical complexes Page S32
Table S22 The most extreme hypothetical complexes Page S33
Table S23 Experimental complexes used for TDDFT benchmark Page S34

Table S24 Correlation between experiment and ANN predictions Page S35
Table S25 Correlation between experiment and TDDFT predictions Page S35
Figure S12 Calculated lifetime vs experiment Page S35
Table S26 Lifetime ANN predictions for complexes with long lifetime Page S36
Figure S13 ANN $Em_{50/50}$ vs TDDFT energy Page S36
Figure S14 ANN lifetime vs TDDFT lifetime Page S37
Figure S15 Confusion matrices for 2 μs lifetime cutoff Page S38
Table S27 Hypothetical complexes used for TDDFT benchmark Page S39
Table S28 Correlation between ANN and TDDFT predictions Page S39
Table S29 70/30 random split $Em_{50/50}$ performance Page S40
Table S30 Dissimilar HLS ligands Page S40
Table S31 Hyperparameters for best ANN models Page S41
Table S32 Exclusion of CSD structures from ligand search Page S41
Figure S16 Phosphor geometry differences for singlet vs triplet state Page S42
Figure S17 TDDFT-calculated energy with CAM-B3LYP Page S43
Figure S18 TDDFT-calculated energy with ωB97X-D3BJ Page S43
References Page S44

Table S1. The labels and structures of the cyclometalating (**CN**) ligands in the HLS. XYZ files of these ligands are provided in the Electronic Supplementary Information .zip file.

**CN1**	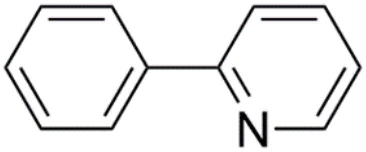	**CN63**	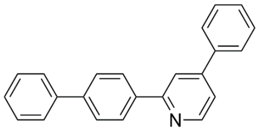
**CN2**	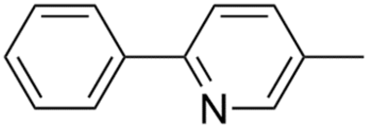	**CN64**	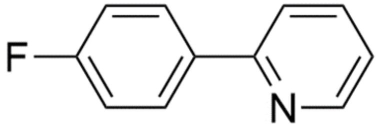
**CN3**	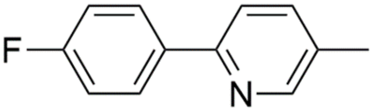	**CN65**	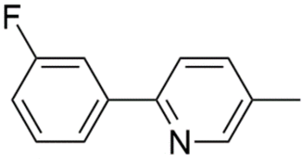
**CN4**	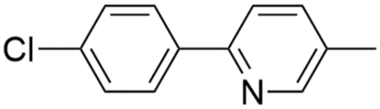	**CN66**	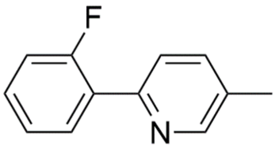
**CN5**	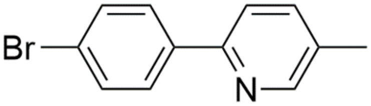	**CN67**	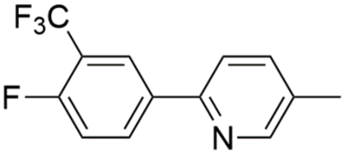
**CN7**	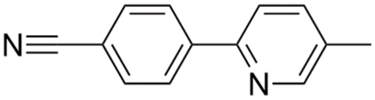	**CN68**	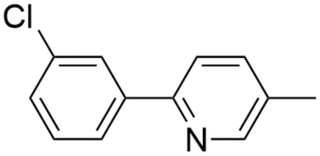
**CN9**	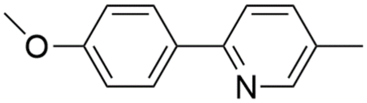	**CN69**	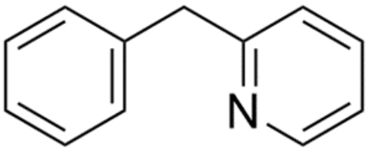
**CN11**	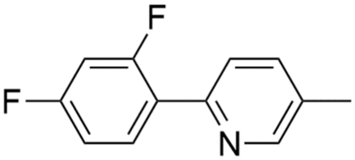	**CN70**	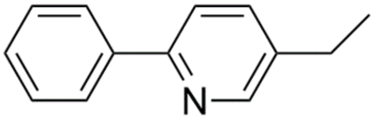

**CN12**	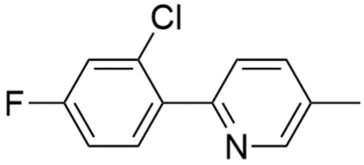	**CN71**	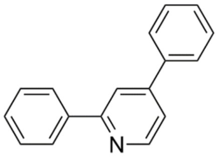
**CN13**	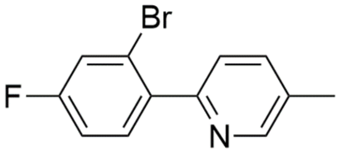	**CN72**	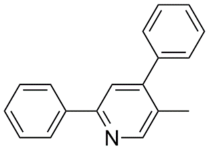
**CN14**	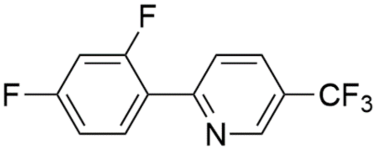	**CN73**	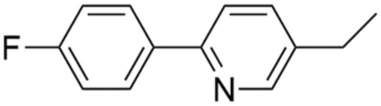
**CN21**	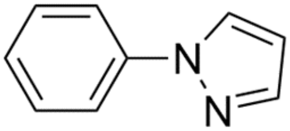	**CN74**	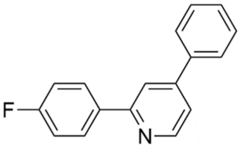
**CN28**	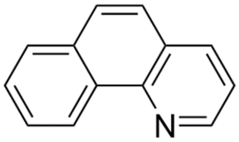	**CN75**	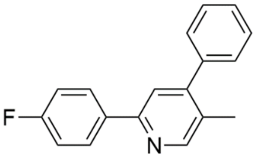
**CN29**	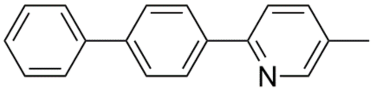	**CN76**	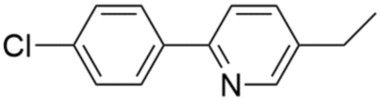
**CN30**	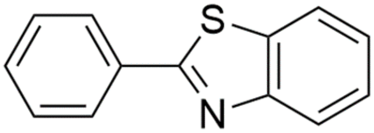	**CN77**	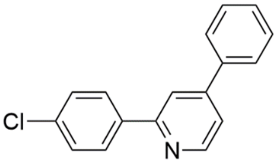
**CN31**	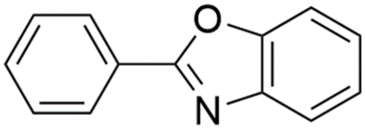	**CN78**	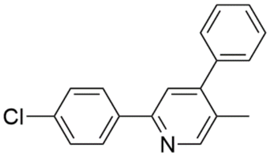

**CN33**	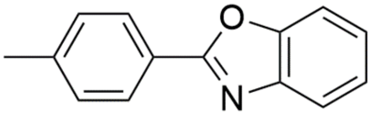	**CN79**	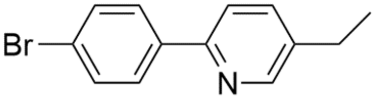
**CN34**	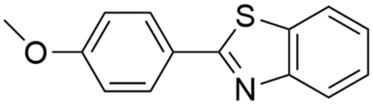	**CN80**	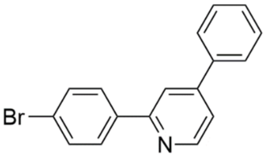
**CN35**	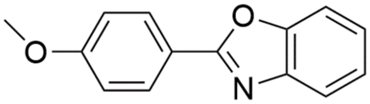	**CN81**	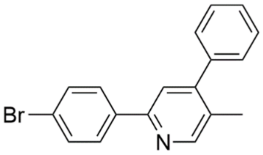
**CN37**	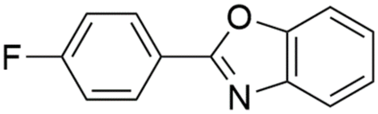	**CN94**	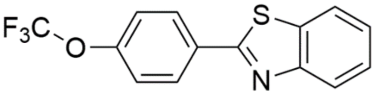
**CN38**	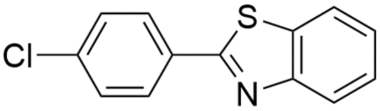	**CN95**	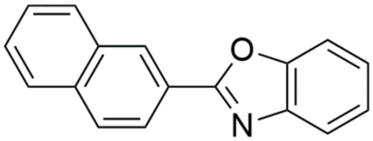
**CN39**	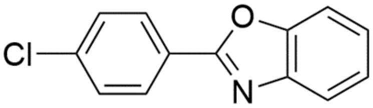	**CN101**	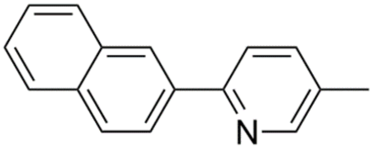
**CN40**	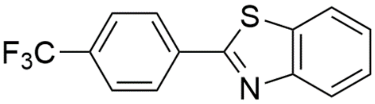	**CN102**	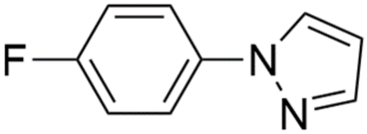
**CN41**	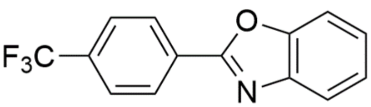	**CN103**	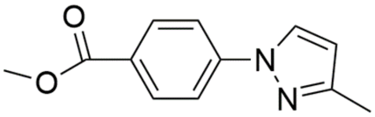

**CN42**	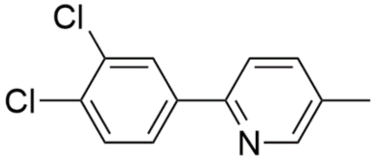	**CN104**	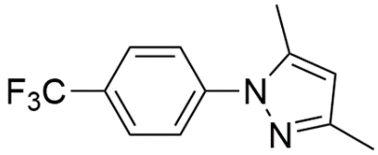
**CN44**	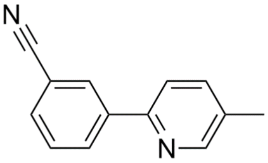	**CN105**	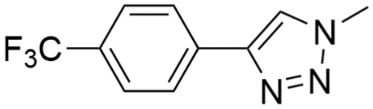
**CN46**	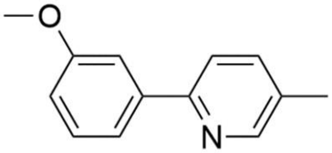	**CN106**	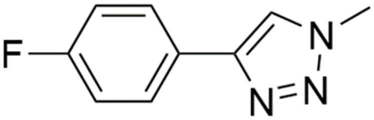
**CN48**	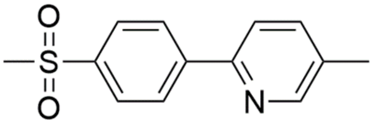	**CN107**	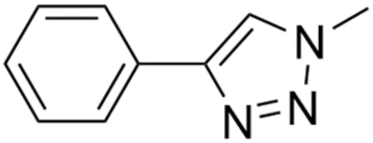
**CN49**	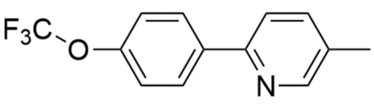	**CN108**	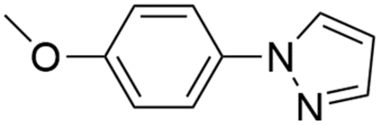
**CN54**	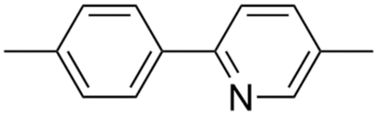	**CN109**	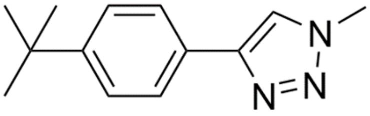

Table S2. The labels and structures of the ancillary (**NN**) ligands in the HLS. XYZ files of these ligands are provided in the Electronic Supplementary Information .zip file. In the .zip file, **NN40**, **NN41**, and **NN42** are in deprotonated form.

**NN1**	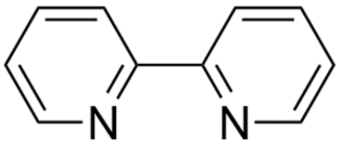	**NN24**	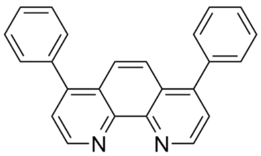
**NN2**	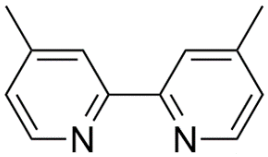	**NN26**	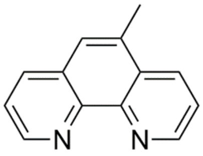
**NN3**	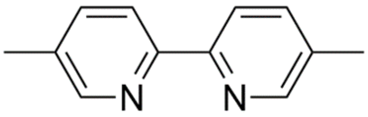	**NN27**	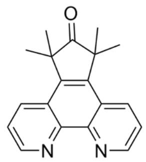
**NN4**	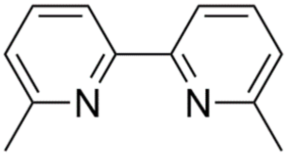	**NN33**	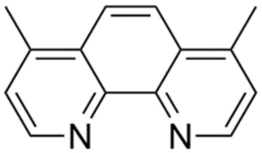
**NN5**	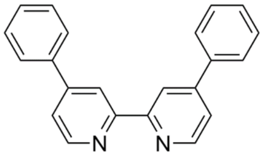	**NN34**	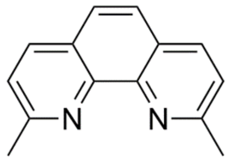
**NN6**	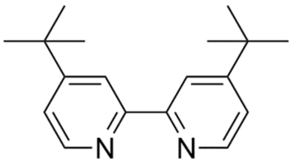	**NN37**	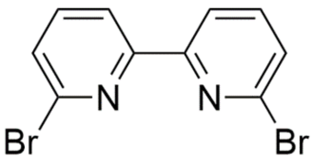
**NN7**	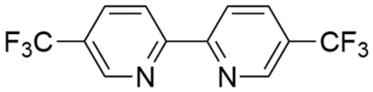	**NN40**	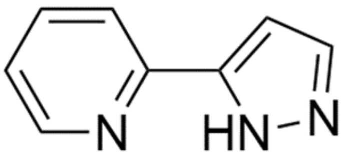
**NN8**	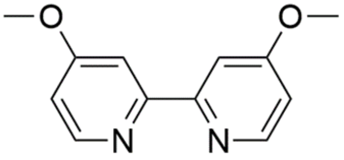	**NN41**	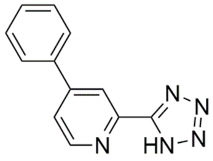

**NN14**	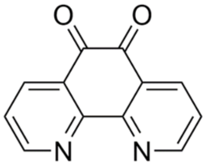	**NN42**	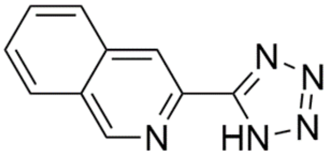
**NN16**	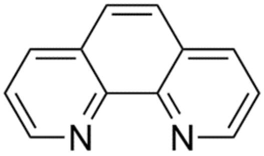	**NN43**	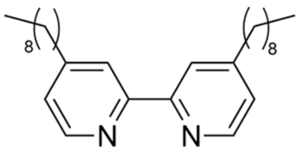
**NN20**	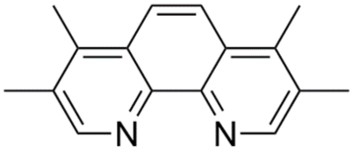	**NN47**	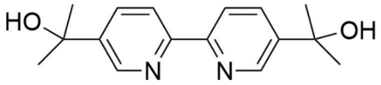
**NN21**	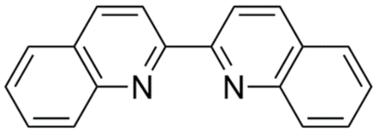		

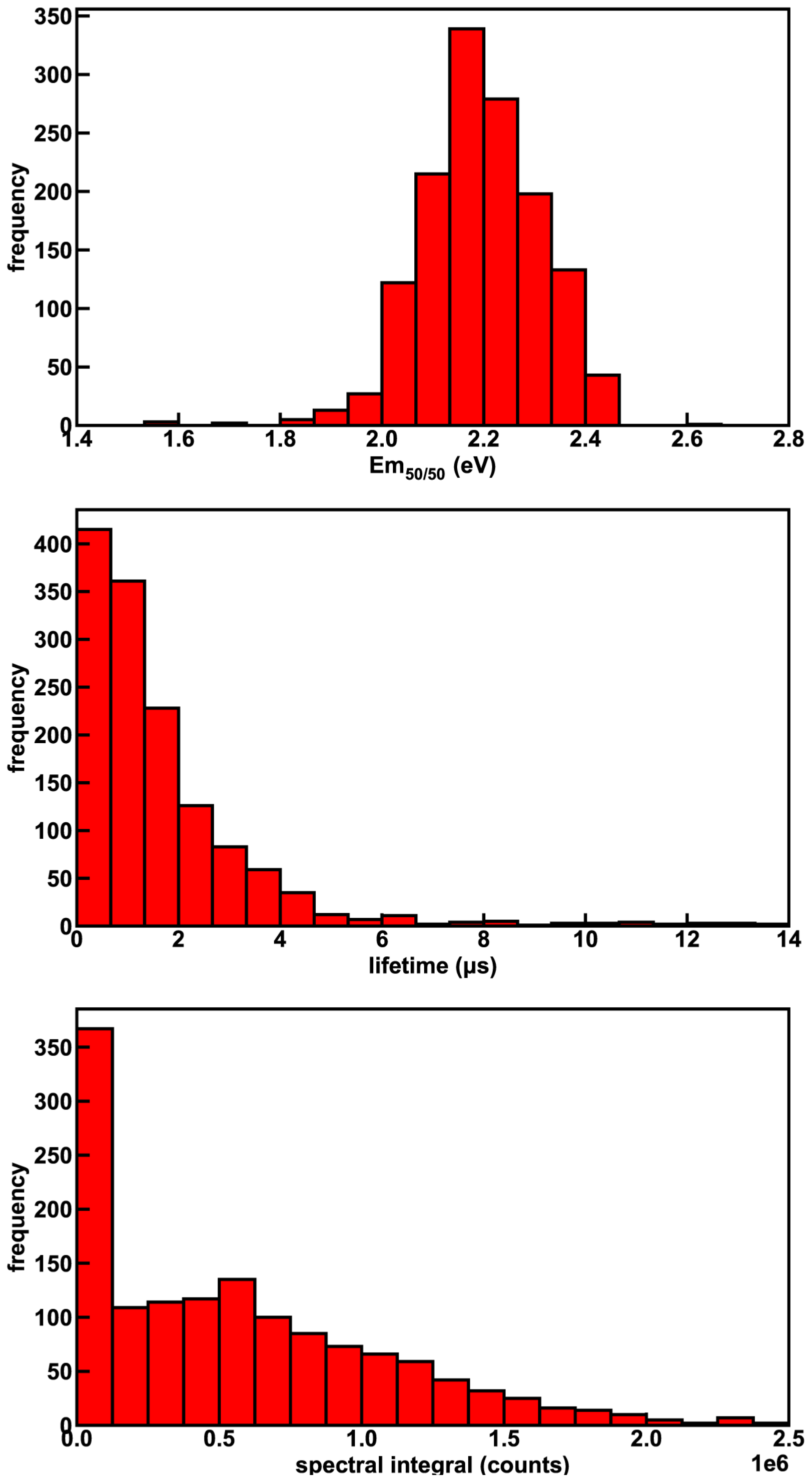

Figure S1. Histograms of the three target properties across the 1,380 complexes reported in the experimental study of DiLuzio *et al.*,[1] excluding the baseline *solvato* complexes that contain a DMSO ligand. The range of phosphorescence lifetimes is restricted to omit eleven outliers with long lifetimes ranging from 14 to 75 μs. Complexes with low spectral integral (less than 1×10^5 photon counts) are considered dim in this work and are excluded from further $Em_{50/50}$ and lifetime analysis (see main text Computational Details).

Text S1. Additional details of calculations and CSD search.

Additional DFT calculation details

As mentioned in the main text, we conducted single-point energy calculations on the optimized neutral ligand geometries at two different charges: +1 and -1. In combination with information from the neutral geometry optimization, this allowed us to calculate vertical IP and EA values analogous to those generated by GFN1-xTB.[2,3] The remaining information (HOMO, LUMO, and Mulliken charges of coordinating atoms) was extracted solely from the neutral geometry optimization calculation for a given ligand. We specified ligands to be in a singlet state when neutral and in a doublet state otherwise.

*Description of **NN40**, **NN41**, and **NN42***

Neutral complexes are generated when **NN40**, **NN41**, or **NN42** is the ancillary ligand. These three ancillary ligands contain tetrazole or pyrazole moieties that deprotonate upon metal coordination.[1] Consequently, we generated these ligands without a hydrogen on the pyrazole/tetrazole nitrogen coordinating atom. As a result, while all other HLS ligands are neutral, these ligands have a charge of -1. We specified this charge for GFN1-xTB calculations, and also adjusted our DFT workflow to account for it as follows: We performed geometry optimization on the **NN40**, **NN41**, and **NN42** ligand geometries generated with Avogadro while specifying a charge of -1. We then conducted single-point energy calculations on the optimized anionic ligand geometries at two different charges: neutral and -2. We specified these ligands to be in a singlet state when at -1 charge and in a doublet state otherwise.

Iridium complexes with these ancillary ligands likely form neutral complexes $[\mathrm{Ir}(\mathbf{CN})_2(\mathbf{NN})]^0$, rather than the +1 charge complexes $[\mathrm{Ir}(\mathbf{CN})_2(\mathbf{NN})]^+$.

Additional CSD details

In a ConQuest search, we searched for structures containing an iridium atom bonded to two carbon atoms and four nitrogen atoms. We set bond types to "Any" and the cyclicity of the carbon and nitrogen atoms to "Cyclic." We searched with 3D coordinates determined and an R factor <= 0.05. Complexes in the hitlist were exported as mol2 files with the "Export largest molecule only" and "One file per entry" options, where the former option removes solvent molecules. From 700 hits, those with multiple iridium atoms and no iridium atoms were removed from consideration, as were hits that still had solvent or counterions, hits that did not have three bidentate ligands, duplicate hits as determined by CSD refcodes, and hits that had a **CC** ligand (Table S32).

Classification of CSD ligands as **CN** or **NN** was accomplished by analyzing the exported mol2 files using molSimplify, which identifies coordinating atoms in addition to identifying ligands. Hydrogen atoms were added to coordinating carbons of CSD **CN** ligands using molSimplify. The presence or absence of a CSD ligand in the HLS was determined through atom-weighted molecular graph determinants, computed using molSimplify. CSD ligands absent from the HLS will be referred to as out-of-HLS CSD ligands. Atom-weighted molecular graph determinants were also used to ensure each out-of-HLS CSD ligand structure was only considered once in hypothetical complex enumeration, since multiple hit complexes might have **CN** or **NN** ligands in common. Hypothetical complexes either had two identical HLS **CN** ligands and an out-of-HLS CSD **NN** ligand, two identical out-of-HLS CSD **CN** ligands and an HLS **NN** ligand, or two identical out-of-HLS CSD **CN** ligands and an out-of-HLS CSD **NN** ligand. From the final complexes in Table S32, 153 unique out-of-HLS **CN** ligands and 269 unique out-of-HLS **NN**

ligands were identified. Consequently, 60,816 hypothetical complexes were considered. Application of uncertainty quantification cutoffs left 70 unique out-of-HLS **CN** ligands and 42 unique out-of-HLS **NN** ligands spread out over 3,598 hypothetical complexes, which we analyzed with our ANNs.

Text S2. Extended description of feature sets.

Explanation of feature notation.

For a given iridium complex, features in the ligand-only RAC set, the xTB set, the B3LYP DFT set, the ωPBEh DFT set, and the Dice set require only the molecular geometry of the **CN** and **NN** ligand of the complex.

For RAC-style feature sets, the atomic properties considered were topology, identity, electronegativity, covalent radius, nuclear charge, group number, and number of bonds by the octet rule, which is also sometimes referred to as the eRAC set when the latter two features are included.[4,5] T is topology, I is identity, χ is electronegativity, S is covalent radius, Z is nuclear charge, Gval is group number, and NumB is number of bonds. For the RAC and CD-RAC feature sets, mc indicates metal-centered, lc indicates ligand-centered, D indicates difference (and its absence indicates product), and depth is indicated by the number in the feature name. The terms all, ax, and eq refer to the extent of a RAC, i.e., whether it spans over the whole complex or only over the axial or equatorial ligands. The four miscellaneous features that describe charge or denticity of a ligand were all removed. For the ligand-only RAC feature set, atom-wise properties, depth, and ligand type are indicated by the feature name.

For the electronic structure feature sets, IP stands for ionization potential and EA stands for electron affinity. The first coordinating nitrogen of the **NN** ligand (N1) is chosen such that the number of nitrogen atoms in its ring is less than or equal to the number of nitrogen atoms in the ring of the second coordinating nitrogen.

Invariant features

Invariant features, i.e., features that are the same across all complexes in the experimental training data, were removed during pre-processing. Only the Morgan, RAC, and CD-RAC feature sets had invariant features.

Morgan and Dice feature sets

A Morgan fingerprint indicates the presence of substructures in a molecule by hashing any given substructure into a X-bit integer, and effectively storing these integers as the indices of bits set to 1 in a 2^X-size bitset.[6] The Morgan feature set initially contains 4,096 features, with 2,048 bits (X=11) allocated to both the **CN** ligand and **NN** ligand; however, over 75% of these features are invariant over the training data and are dropped from the set. The Dice coefficient between two Morgan fingerprints A and B is defined as $\frac{2c}{a+b}$, where a is the number of bits set to 1 in A, b is the number of bits set to 1 in B, and c is the number of bits set to 1 in both A and B.[7] We also considered the popular Tanimoto[7] coefficients ($\frac{c}{a+b-c}$) but found these to hold less predictive power than Dice coefficients in the present application.

Revised autocorrelation functions details

RAC features can be full-scope, metal-centered, or ligand-centered; these distinctions indicate the positions of the starting atoms used to generate the RAC feature. For a full-scope RAC feature, every atom in the complex can be used as the starting atom. For a metal-centered RAC feature, the metal center serves as the starting atom. For a ligand-centered RAC feature, the coordinating atoms on the ligands serve as starting atoms.

Mathematically, autocorrelations are defined as

$$P_d = \Sigma_i \ \Sigma_j \ P_i P_j \delta(d_{ij}, d) \quad (1)$$

$$P'_d = \Sigma_i \ \Sigma_j \ (P_i - P_j) \delta(d_{ij}, d) \quad (2)$$

where P_d is the graph autocorrelation for property P at depth d, P'_d is the analogous difference graph autocorrelation, P_i is the property P for atom i, δ is the Dirac delta function, and d_{ij} is the bond-wise path distance between atoms i and j.

For CD-RACs,[8] there is an adjustment for the spatial distance between two atoms. CD-RACs are defined as

$$P_{d,CD} = \frac{1}{n} \begin{cases} \Sigma_i \ \Sigma_j \ \dfrac{P_i P_j}{r_{ij}} \delta\left(d_{ij}, d\right), & d > 0 \\ \dfrac{1}{2} \Sigma_i \ P_i^{2.4}, & d = 0 \end{cases} \quad (3)$$

where $P_{d,CD}$ is the Coulomb-decay graph autocorrelation for property P at depth d, n is the number of atoms, P_i is the property P for atom i, r_{ij} is the spatial distance between atoms i and j, δ is the Dirac delta function, and d_{ij} is the bond-wise path distance between atoms i and j. Difference CD-RACs are analogously defined.

RAC-style feature sets details

For the RAC feature set, we allowed *d* to range from zero – corresponding to the correlation of an atom property to itself – to three. The RAC feature set consists of 196 features.

For the ligand-only RAC feature set, we allowed *d* to range from zero to four. We used a larger maximum depth than that of the RAC feature set due to the comparatively low number of features in the ligand-only case. This feature set was motivated by our anticipation that separate treatment of **CN** and **NN** ligands would improve predictive power. Furthermore, the absence of metal-centered information in this feature set is acceptable because it was anticipated that the metal-centered RACs in the RAC feature set would be uninformative - all complexes in this study have an iridium metal center and an identical first coordination sphere of two carbon atoms and four nitrogen atoms. The ligand-only RAC feature set consists of 70 features total.

For the CD-RAC feature set, we allowed *d* to range from zero to three. Both the RAC feature set and the ligand-only RAC feature sets can be generated from structures that are not geometry optimized. This is because RACs are connectivity-dependent features but do not depend on geometry information such as bond lengths. In contrast, CD-RACs are affected by geometry optimization. CD-RACs were calculated on molSimplify-generated structures optimized with UFF. This feature set was motivated by the impact of bond distances on Ir phosphor properties like quantum yield.[9] The CD-RAC feature set consists of 222 features.

Table S3. The eighty-three features in the Dice feature set obtained over a random split of the test data. Features and notation are described in detail in Text S2. For the grouped split of the train/test data, there are five fewer features in the Dice feature set due to the absence of five HLS ligands from the training data.

feature type	feature count	feature list
Dice coefficient between the Morgan fingerprint of the **CN** ligand of the current complex and the Morgan fingerprint of an HLS **CN** ligand	60	similarity to **CN1**<br>similarity to **CN2**<br>similarity to **CN3**<br>…<br>similarity to **CN109**
Dice coefficient between the Morgan fingerprint of the **NN** ligand of the current complex and the Morgan fingerprint of an HLS **NN** ligand	23	similarity to **NN1**<br>similarity to **NN2**<br>similarity to **NN3**<br>…<br>similarity to **NN47**

Table S4. Comparison of ANN performance on a random split of the test data, as measured by the mean absolute errors of model test set predictions, using the Dice feature set versus using an analogous Tanimoto feature set. We used RDKit 2021.9.2[10] to generate Morgan fingerprints of ligands and Tanimoto similarity coefficients in the same way Dice similarity coefficients were generated (see main text *Feature Sets* discussion).

	target property		
	$Em_{50/50}$ (eV)	lifetime (μs)	spectral integral (photon counts)
Dice	0.0161	0.7540	1.22E5
Tanimoto	0.0169	0.8381	1.31E5

Table S5. The 196 features in the eRAC[5] feature set. Each category of features contains twenty-eight features to start (i.e., seven atomic properties at four depths, 0, 1, 2, or 3). Invariant features that are removed are listed below along with a final feature count. Features and notation are described in detail in Text S2.

start	extent	operation	features removed	feature count
full-scope	all	product	0	28
	axial ligand	product	0	28
	equatorial ligand	product	0	28
metal-centered	all	product	(14) mc-chi-0-all, mc-Z-0-all, mc-Z-1-all, mc-I-0-all, mc-I-1-all, mc-I-2-all, mc-T-0-all, mc-T-1-all, mc-S-0-all, mc-S-1-all, mc-Gval-0-all, mc-Gval-1-all, mc-NumB-0-all, mc-NumB-1-all	14
		difference	(16) D_mc-chi-0-all, D_mc-chi-1-all, D_mc-Z-0-all, D_mc-Z-1-all, D_mc-I-0-all, D_mc-I-1-all, D_mc-I-2-all, D_mc-I-3-all, D_mc-T-0-all, D_mc-T-1-all, D_mc-S-0-all, D_mc-S-1-all, D_mc-Gval-0-all, D_mc-Gval-1-all, D_mc-NumB-0-all, D_mc-NumB-1-all	12
ligand-centered	axial ligand	product	(3) lc-I-0-ax, lc-I-1-ax, lc-T-0-ax	25
		difference	(10) D_lc-chi-0-ax, D_lc-Z-0-ax, D_lc-I-0-ax, D_lc-I-1-ax, D_lc-I-2-ax, D_lc-I-3-ax, D_lc-T-0-ax, D_lc-S-0-ax, D_lc-Gval-0-ax, D_lc-NumB-0-ax	18
	equatorial ligand	product	(3) lc-I-0-eq, lc-I-1-eq, lc-T-0-eq	25
		difference	(10) D_lc-chi-0-eq, D_lc-Z-0-eq, D_lc-I-0-eq, D_lc-I-1-eq, D_lc-I-2-eq, D_lc-I-3-eq, D_lc-T-0-eq, D_lc-S-0-eq, D_lc-Gval-0-eq, D_lc-NumB-0-eq	18

Table S6. The seventy features in the ligand-only eRAC feature set. Each combination of ligand and atomic property yields five features due to depths ranging from 0 to 4. Features and notation are described in detail in Text S2.

ligand	atomic property	feature list
**CN**	topology	T-0_CN, T-1_CN, T-2_CN, T-3_CN, T-4_CN
	identity	I-0_CN, I-1_CN, I-2_CN, I-3_CN, I-4_CN
	electronegativity	chi-0_CN, chi-1_CN, chi-2_CN, chi-3_CN, chi-4_CN
	covalent radius	S-0_CN, S-1_CN, S-2_CN, S-3_CN, S-4_CN
	nuclear charge	Z-0_CN, Z-1_CN, Z-2_CN, Z-3_CN, Z-4_CN
	group number	Gval-0_CN, Gval-1_CN, Gval-2_CN, Gval-3_CN, Gval-4_CN
	number of bonds	NumB-0_CN, NumB-1_CN, NumB-2_CN, NumB-3_CN, NumB-4_CN
**NN**	topology	T-0_NN, T-1_NN, T-2_NN, T-3_NN, T-4_NN
	identity	I-0_NN, I-1_NN, I-2_NN, I-3_NN, I-4_NN
	electronegativity	chi-0_NN, chi-1_NN, chi-2_NN, chi-3_NN, chi-4_NN
	covalent radius	S-0_NN, S-1_NN, S-2_NN, S-3_NN, S-4_NN
	nuclear charge	Z-0_NN, Z-1_NN, Z-2_NN, Z-3_NN, Z-4_NN
	group number	Gval-0_NN, Gval-1_NN, Gval-2_NN, Gval-3_NN, Gval-4_NN
	number of bonds	NumB-0_NN, NumB-1_NN, NumB-2_NN, NumB-3_NN, NumB-4_NN

Table S7. The 222 features in the CD-RAC feature set. Each category of features contains twenty-eight features to start (i.e., seven atomic properties at four depths, 0, 1, 2, or 3). Invariant features that are removed are listed below along with a final feature count. The notation of features and property type is the same as in RACs but incorporates a distance-dependent term as outlined in ref. [8]. Features and notation are described in detail in Text S2.

start	extent	operation	features removed	feature count
full-scope	all	product	0	28
	axial ligand	product	0	28
	equatorial ligand	product	0	28
metal-centered	all	product	0	28
		difference	(10) D_mc-chi-0-all, D_mc-Z-0-all, D_mc-I-0-all, D_mc-I-1-all, D_mc-I-2-all, D_mc-I-3-all, D_mc-T-0-all, D_mc-S-0-all, D_mc-Gval-0-all, D_mc-NumB-0-all	18
ligand-centered	axial ligand	product	0	28
		difference	(10) D_lc-chi-0-ax, D_lc-Z-0-ax, D_lc-I-0-ax, D_lc-I-1-ax, D_lc-I-2-ax, D_lc-I-3-ax, D_lc-T-0-ax, D_lc-S-0-ax, D_lc-Gval-0-ax, D_lc-NumB-0-ax	18
	equatorial ligand	product	0	28
		difference	(10) D_lc-chi-0-eq, D_lc-Z-0-eq, D_lc-I-0-eq, D_lc-I-1-eq, D_lc-I-2-eq, D_lc-I-3-eq, D_lc-T-0-eq, D_lc-S-0-eq, D_lc-Gval-0-eq, D_lc-NumB-0-eq	18

Table S8. The twelve features present in each of the xTB, B3LYP DFT, and ωPBEh DFT feature sets (i.e., evaluated at each level of theory).

feature type	ligand	feature count	feature list
energy levels	**CN**	2	HOMO of **CN** ligand<br>LUMO of **CN** ligand
	**NN**	2	HOMO of **NN** ligand<br>LUMO of **NN** ligand
energy descriptor	**CN**	2	IP of **CN** ligand<br>EA of **CN** ligand
	**NN**	2	IP of **NN** ligand<br>EA of **NN** ligand
Mulliken charges	**CN**	2	charge of coordinating carbon<br>charge of coordinating nitrogen
	**NN**	2	charge of first coordinating nitrogen<br>charge of second coordinating nitrogen

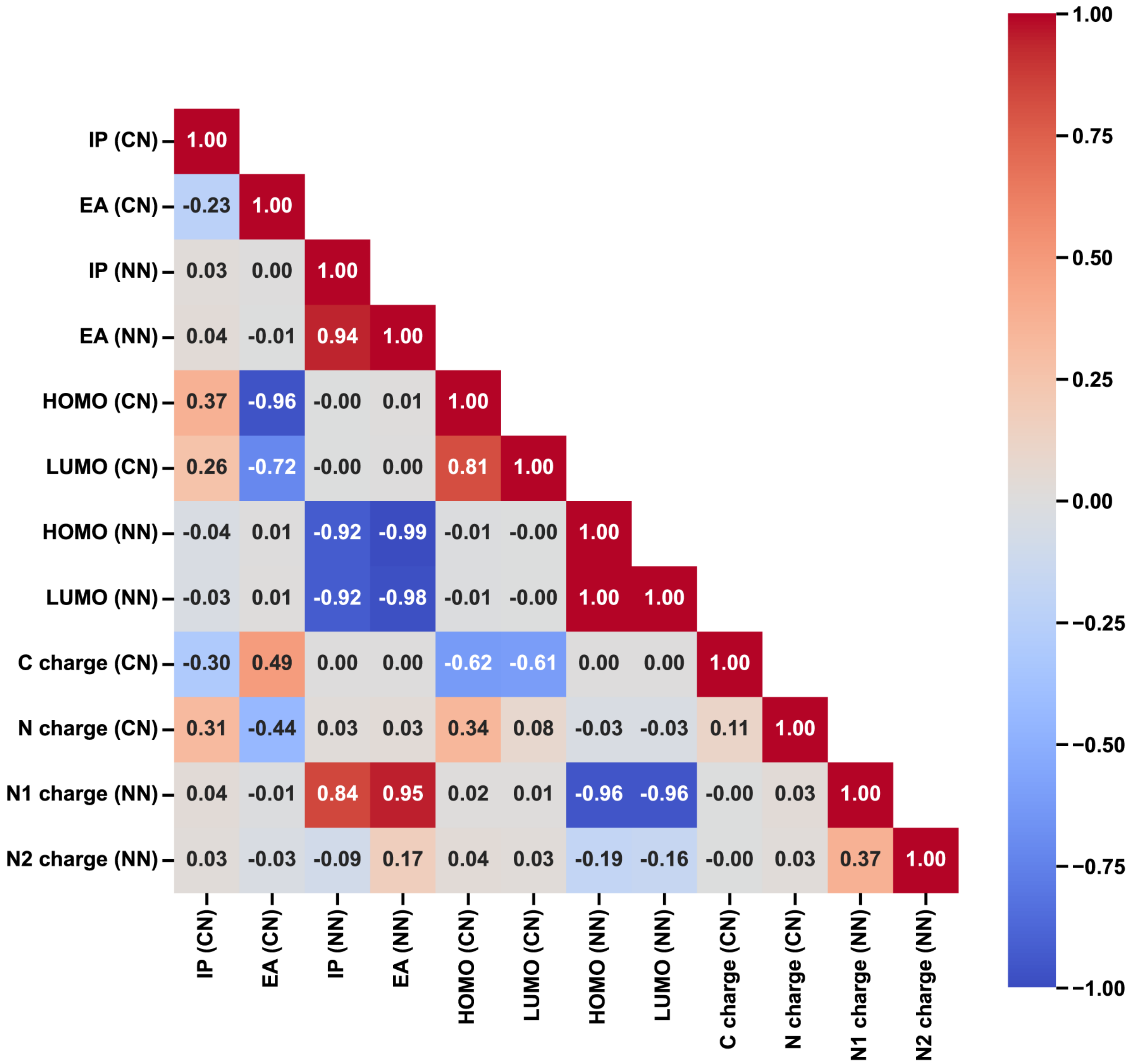

Figure S2. The signed Pearson correlation coefficients (-1 in blue to +1 in red with gray for 0 as indicated in the colorbar on the right) of the xTB features with each other across the original dataset in the experimental study of DiLuzio *et al.*,[1] excluding the baseline *solvato* complexes that contain a DMSO ligand and complexes with a spectral integral below 10^5 photon counts.

Table S9. Comparison of random split ANN performance, as measured by the mean absolute errors of model test set predictions, using natural charges instead of Mulliken charges for the connecting atom charge features of the B3LYP DFT and ωPBEh DFT feature sets. Although Mulliken charges lead to worse performance on the random split, they lead to better performance on the grouped split. Regardless of which charge scheme is used for the DFT feature sets, the xTB feature set outperforms the B3LYP DFT and ωPBEh DFT feature sets.

		target property		
		$Em_{50/50}$ (eV)	lifetime (μs)	spectral integral (photon counts)
B3LYP DFT	Mulliken charges	0.0290	1.0105	1.81E5
	natural charges	0.0251	1.0134	1.75E5
ωPBEh DFT	Mulliken charges	0.0285	0.9550	1.92E5
	natural charges	0.0233	0.9659	1.69E5

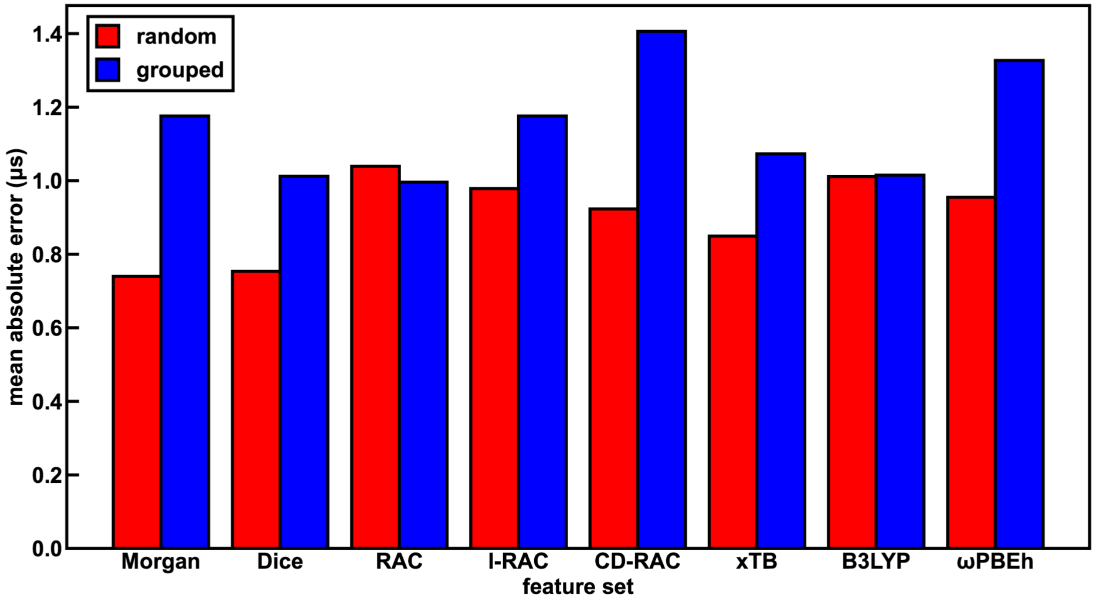

Figure S3. The test set performance of ANNs trained on different feature sets in predicting lifetime (in units of μs) for both random (red bars) and grouped splits (blue bars). The Morgan feature set leads to the best performance on the random split, and the RAC feature set leads to the best performance on the grouped split.

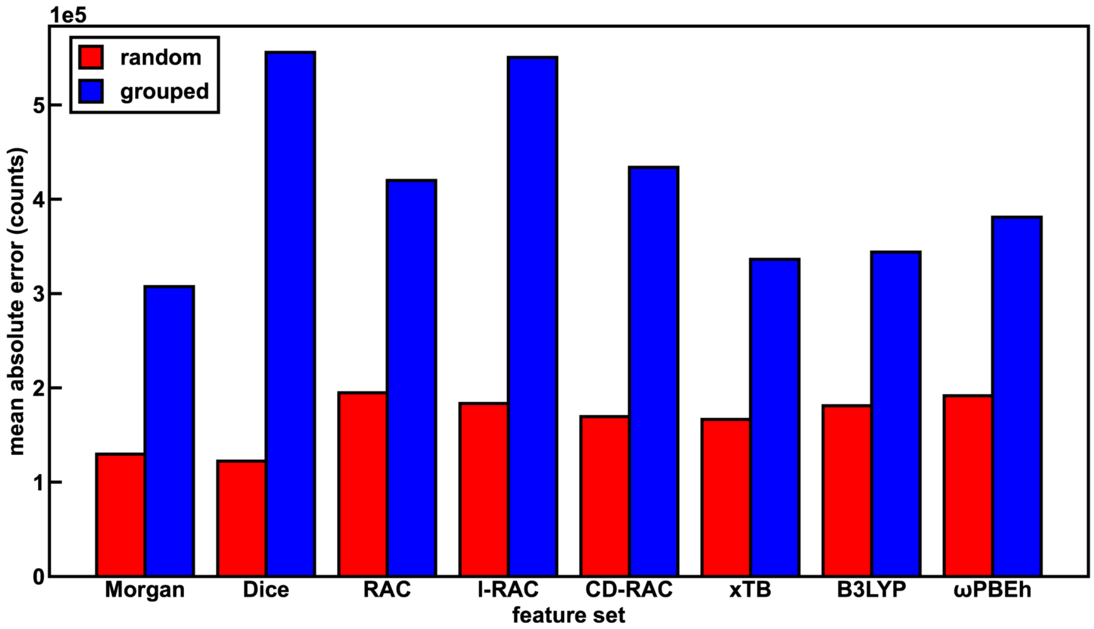

Figure S4. The test set performance of ANNs trained on different feature sets in predicting spectral integral (in units of photon counts) for both random (red bars) and grouped splits (blue bars). The Dice feature set leads to the best performance on the random split, and the Morgan feature set leads to the best performance on the grouped split.

Table S10. The ranking of feature sets for ANNs trained and tested on a random split using different feature sets in predicting each of the target properties. The ranking is on the basis of test set MAE, and a rank of 1 indicates the corresponding feature set led to the ANN with the lowest MAE for the specified target property.

	$Em_{50/50}$ rank	lifetime rank	spectral integral rank	mean rank
Dice	1	2	1	1.33
Morgan	2	1	2	1.67
xTB	3	3	3	3.00
CD-RAC	5	4	4	4.33
ligand-only RAC	4	6	6	5.33
ωPBEh DFT	7	5	7	6.33
B3LYP DFT	8	7	5	6.67
RAC	6	8	8	7.33

Table S11. The MAE, scaled MAE, and R^2 test set performance of the eight random split ANNs predicting for $Em_{50/50}$. Scaled MAE is defined as MAE divided by the difference between the maximum and minimum value of the target property in the training data.

	MAE (eV)	scaled MAE	R^2
Morgan	0.0163	0.031	0.91
Dice	0.0161	0.031	0.92
RAC	0.0274	0.053	0.83
ligand-only RAC	0.0220	0.042	0.89
CD-RAC	0.0233	0.045	0.87
xTB	0.0210	0.040	0.91
B3LYP DFT	0.0290	0.056	0.83
ωPBEh DFT	0.0285	0.055	0.85

Table S12. The MAE, scaled MAE, and R^2 test set performance of the eight random split ANNs predicting for lifetime.

	MAE (μs)	scaled MAE	R^2
Morgan	0.7397	0.045	0.36
Dice	0.7540	0.046	0.38
RAC	1.0389	0.064	0.26
ligand-only RAC	0.9789	0.060	0.20
CD-RAC	0.9233	0.057	0.32
xTB	0.8495	0.052	0.38
B3LYP DFT	1.0105	0.062	0.29
ωPBEh DFT	0.9550	0.059	0.33

Table S13. The MAE, scaled MAE, and R^2 test set performance of the eight random split ANNs predicting for spectral integral.

	MAE (photon counts)	scaled MAE	R^2
Morgan	1.30E+05	0.054	0.84
Dice	1.22E+05	0.050	0.87
RAC	1.95E+05	0.080	0.73
ligand-only RAC	1.83E+05	0.075	0.73
CD-RAC	1.70E+05	0.070	0.79
xTB	1.67E+05	0.068	0.79
B3LYP DFT	1.81E+05	0.074	0.77
ωPBEh DFT	1.92E+05	0.079	0.72

Table S14. The Pearson correlation coefficient for the analogous electronic structure features in the xTB feature set and the B3LYP DFT and ωPBEh DFT feature sets. The correlations are evaluated across the original dataset in the experimental study of DiLuzio *et al.*,[1] excluding the baseline *solvato* complexes that contain a DMSO ligand.

	xTB vs B3LYP	xTB vs ωPBEh
IP (**CN**)	0.93	0.50
EA (**CN**)	0.96	0.93
IP (**NN**)	1.00	0.90
EA (**NN**)	0.99	0.98
HOMO (**CN**)	0.13	0.05
LUMO (**CN**)	0.56	0.55
HOMO (**NN**)	0.94	0.86
LUMO (**NN**)	0.91	0.93
C charge (**CN**)	-0.19	-0.13
N charge (**CN**)	0.86	0.86
N1 charge (**NN**)	-0.05	0.05
N2 charge (**NN**)	-0.15	-0.06

Table S15. The MAE, scaled MAE, and R^2 test set performance of the eight grouped split ANNs predicting for $Em_{50/50}$.

	MAE (eV)	scaled MAE	R^2
Morgan	0.0522	0.100	0.41
Dice	0.0722	0.138	-0.27
RAC	0.0547	0.105	0.46
ligand-only RAC	0.0646	0.124	0.17
CD-RAC	0.0663	0.127	0.28
xTB	0.0410	0.078	0.70
B3LYP DFT	0.0600	0.115	0.32
ωPBEh DFT	0.0464	0.089	0.59

Table S16. The MAE, scaled MAE, and R^2 test set performance of the eight grouped split ANNs predicting for lifetime.

	MAE (μs)	scaled MAE	R^2
Morgan	1.1760	0.049	0.06
Dice	1.0120	0.043	0.36
RAC	0.9960	0.042	0.22
ligand-only RAC	1.1759	0.049	0.26
CD-RAC	1.4060	0.059	-0.05
xTB	1.0730	0.045	0.21
B3LYP DFT	1.0149	0.043	0.26
ωPBEh DFT	1.3272	0.056	0.06

Table S17. The MAE, scaled MAE, and R^2 test set performance of the eight grouped split ANNs predicting for spectral integral.

	MAE (photon counts)	scaled MAE	R^2
Morgan	3.07E+05	0.127	0.23
Dice	5.56E+05	0.229	-1.51
RAC	4.20E+05	0.173	-0.48
ligand-only RAC	5.50E+05	0.226	-1.16
CD-RAC	4.34E+05	0.178	-0.66
xTB	3.36E+05	0.138	0.08
B3LYP DFT	3.44E+05	0.141	-0.04
ωPBEh DFT	3.81E+05	0.157	-0.23

Table S18. The percent change in test set MAE for each ANN feature set from the random split to the grouped split.

	$Em_{50/50}$	lifetime	spectral integral
xTB	95	26	102
Morgan	220	59	137
RAC	99	-4	115
B3LYP DFT	107	0	90
ωPBEh DFT	63	39	99
ligand-only RAC	194	20	200
Dice	348	34	355
CD-RAC	185	52	156
average	164	28	157

Table S19. The ranking of grouped split ANNs using different feature sets in predicting for each of the target properties. The ranking is on the basis of test set MAE, and a rank of 1 indicates the corresponding feature set led to the ANN with the lowest MAE for the specified target property.

	$Em_{50/50}$ rank	lifetime rank	spectral integral rank	mean rank
xTB	1	4	2	2.33
Morgan	3	6	1	3.33
RAC	4	1	5	3.33
B3LYP DFT	5	3	3	3.67
ωPBEh DFT	2	7	4	4.33
ligand-only RAC	6	5	7	6.00
Dice	8	2	8	6.00
CD-RAC	7	8	6	7.00

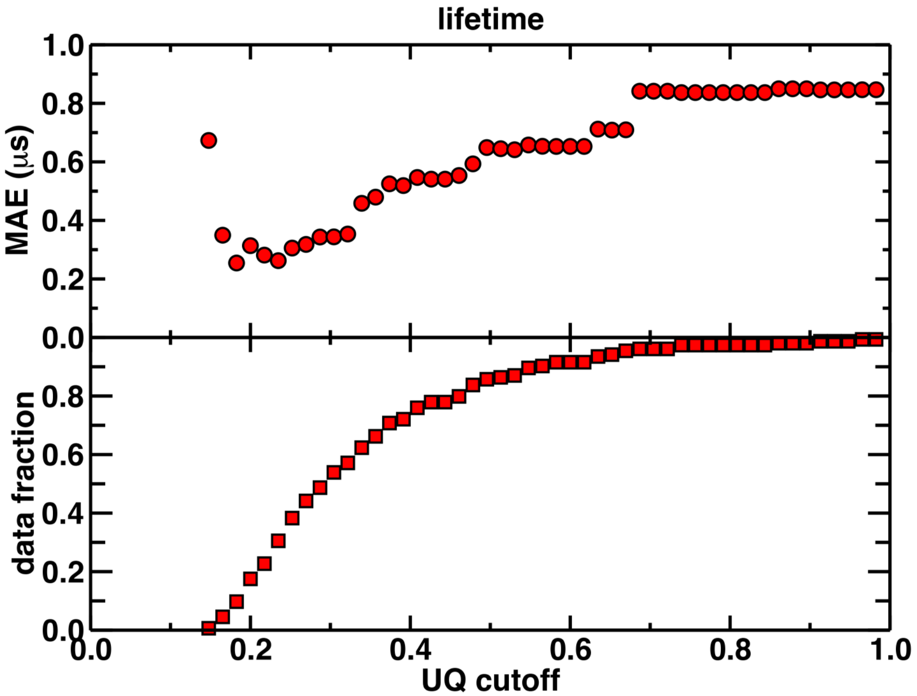

Figure S5. The uncertainty quantification (UQ) cutoff versus test set mean absolute error (in μs) and data fraction of the random split ANN model trained on the xTB feature set and predicting lifetime. The data fraction is the number of test set complexes under the corresponding UQ cutoff, and the MAE is calculated on this subset of complexes. The UQ metric used is the average latent space distance to the ten nearest neighbors in the training set following the protocol introduced in Ref. 11. The UQ metric is normalized such that the largest UQ metric is scaled to 1.

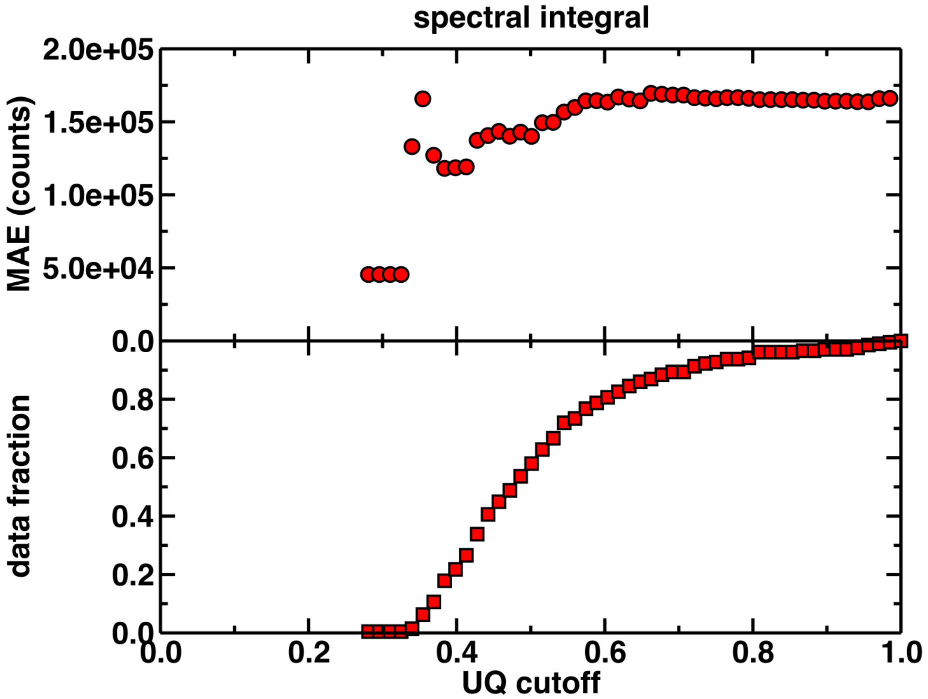

Figure S6. The uncertainty quantification (UQ) cutoff versus test set mean absolute error (in photon counts) of the random split ANN model trained on the xTB feature set and predicting spectral integral. The data fraction is the number of test set complexes under the corresponding UQ cutoff, and the MAE is calculated on this subset of complexes. The UQ metric used is the average latent space distance to the ten nearest neighbors in the training set following the protocol introduced in Ref. 11. The UQ metric is normalized such that the largest UQ metric is scaled to 1.

Table S20. The random split test set performance of a default sklearn linear model with L2 regularization, a sklearn random forest regressor, and an ANN in predicting for each target property. All models were provided with xTB features.

		MAE	R^2
$Em_{50/50}$	linear	0.0683	0.27
	random forest	0.0317	0.80
	ANN	0.0210	0.91
lifetime	linear	1.2254	0.15
	random forest	0.8148	0.46
	ANN	0.8495	0.38
spectral integral	linear	3.68E+05	0.26
	random forest	1.80E+05	0.76
	ANN	1.67E+05	0.79

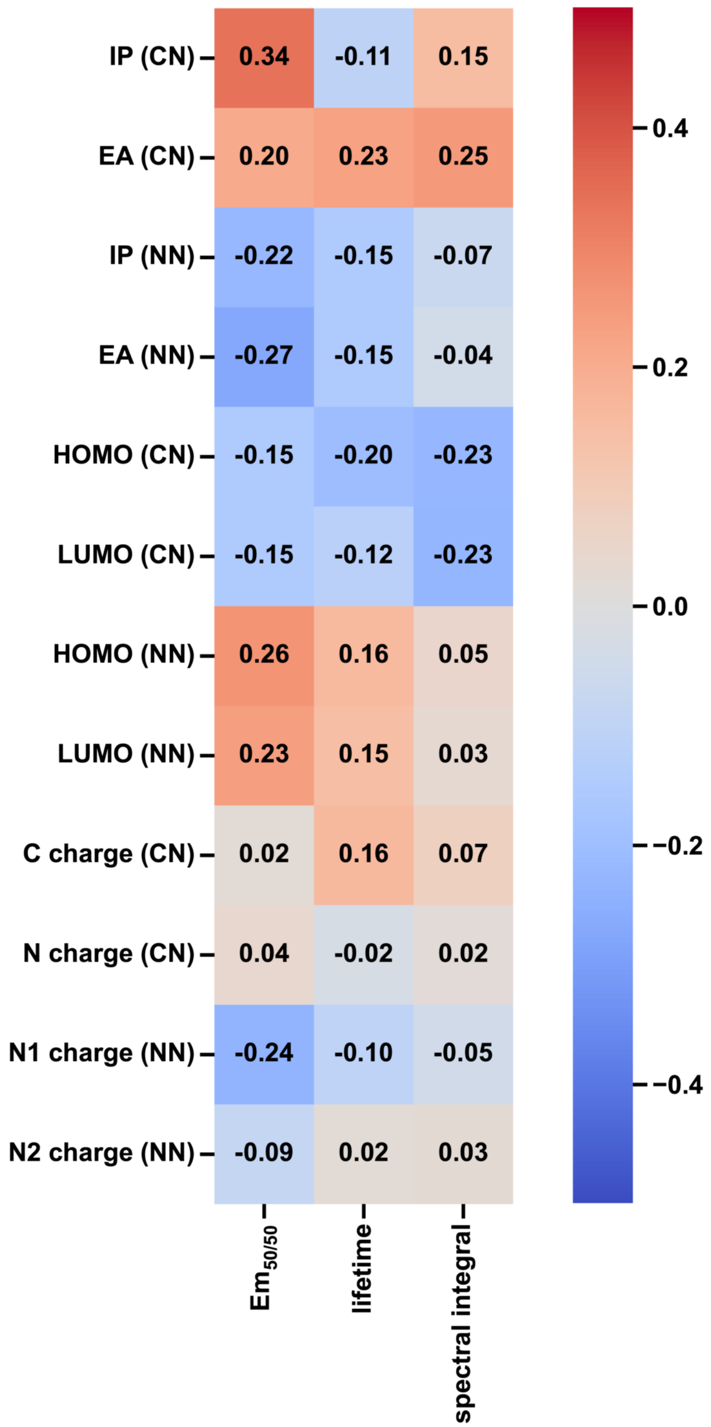

Figure S7. The signed Pearson correlation coefficients (-0.5 in blue to +0.5 in red with gray for 0 as indicated in the colorbar on the right) of the xTB features with the phosphor properties across the original dataset in the experimental study of DiLuzio *et al.*,[1] excluding the baseline *solvato* complexes that contain a DMSO ligand and complexes with a spectral integral below 10^5 photon counts.

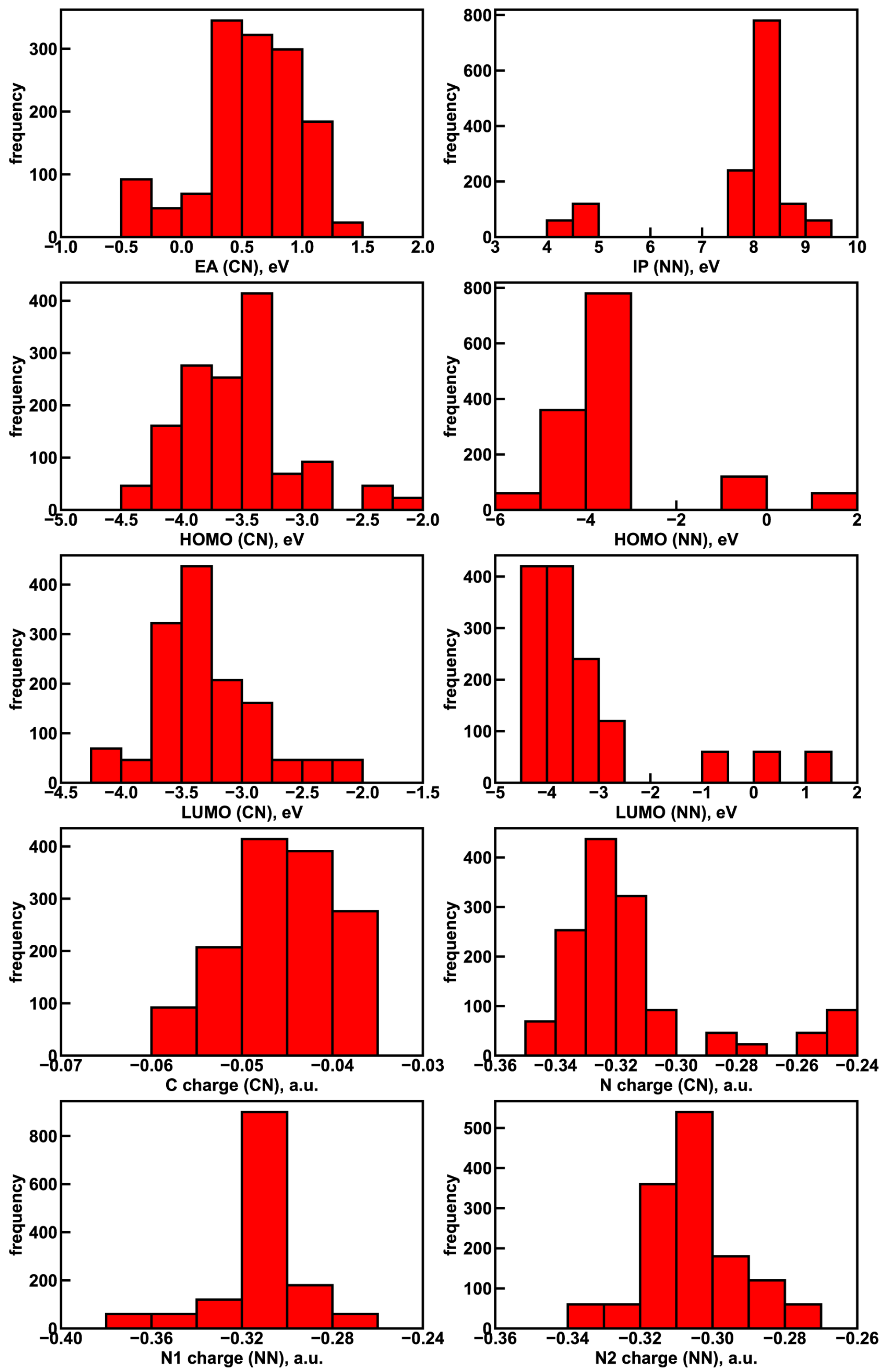

Figure S8. xTB feature distributions over the 1,380 complexes reported in the experimental study of DiLuzio *et al.*,[1] excluding the baseline *solvato* complexes.

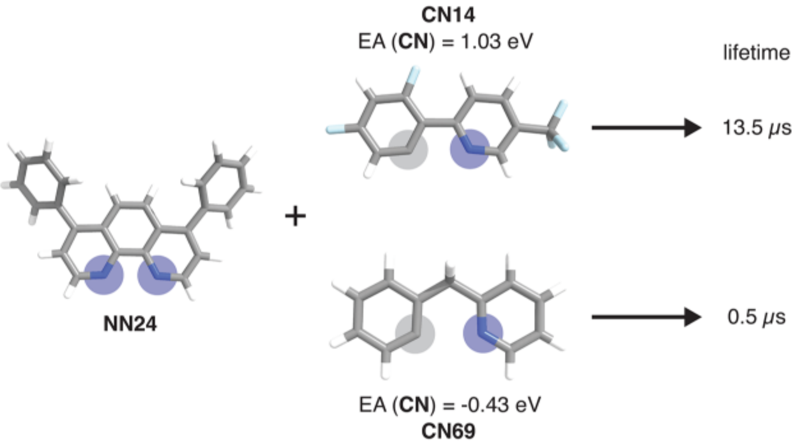

Figure S9. Example of a pair of complexes where the substitution of the **CN** ligand leads to a large lifetime property change. Here, complexes are represented by the combination of a **CN** and **NN** ligand. Coordinated nitrogen (carbon) atoms are indicated with blue (gray) circles. The relevant xTB features for the substituted ligands are shown. Atoms are colored as follows: white for hydrogen, gray for carbon, blue for nitrogen, and light blue for fluorine.

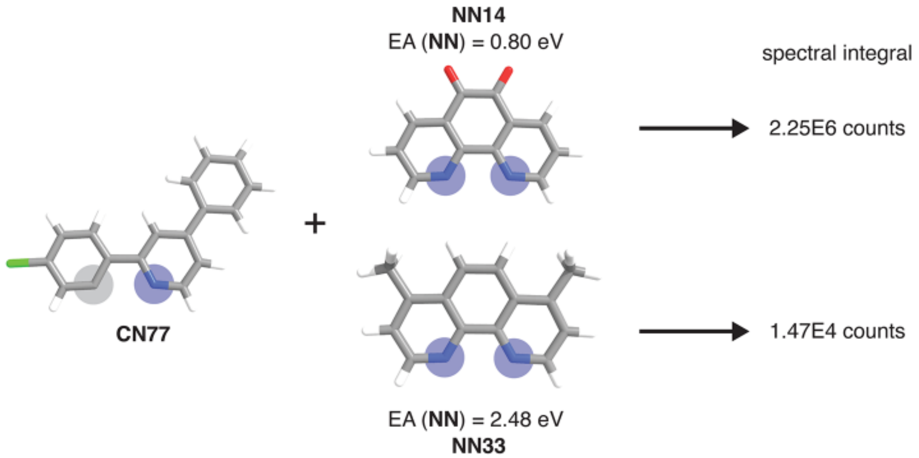

Figure S10. Example of a pair of complexes where the substitution of the **NN** ligand leads to a large spectral integral property change. Here, complexes are represented by the combination of a **CN** and **NN** ligand. Coordinated nitrogen (carbon) atoms are indicated with blue (gray) circles. The relevant xTB features for the substituted ligands are shown. Atoms are colored as follows: white for hydrogen, gray for carbon, blue for nitrogen, red for oxygen, and green for chlorine.

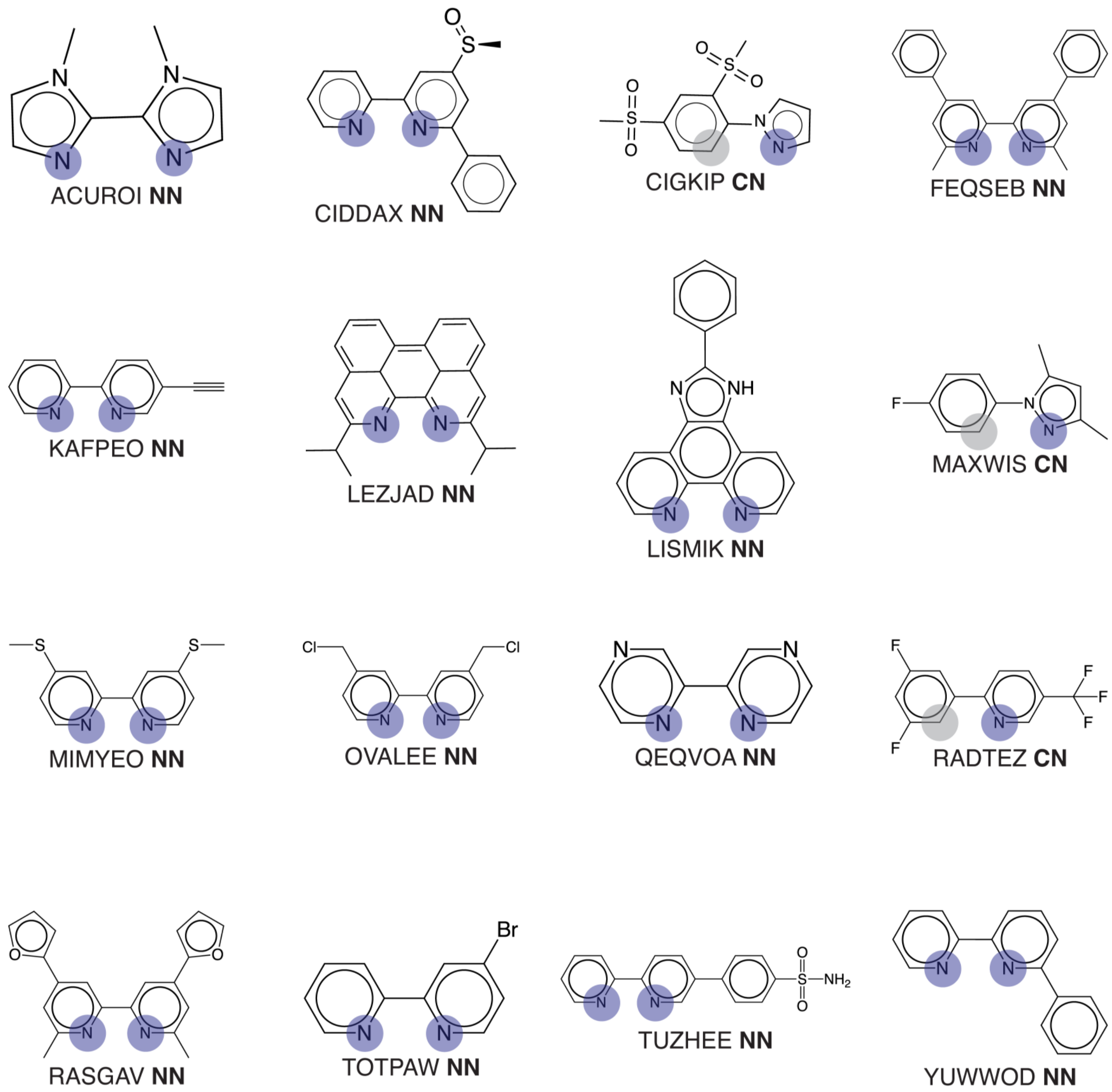

Figure S11. Sixteen CSD ligands that lead to extreme predicted phosphor properties. The six-letter identifiers are CSD refcodes of each complex from which the ligand was extracted. Coordinated nitrogen (carbon) atoms are indicated with blue (gray) circles. ACUROI **NN** appears frequently in hypothetical complexes with high predicted spectral integral. CIDDAX **NN** is present in the hypothetical complex with the lowest predicted lifetime. CIGKIP **CN** is present in the hypothetical complex with the highest predicted spectral integral. FEQSEB **NN** appears frequently in hypothetical complexes with high predicted $Em_{50/50}$ and lifetime. KAFPEO **NN** appears frequently in hypothetical complexes with low predicted spectral integral. LEZJAD **NN** is present in two of the hypothetical complexes with the lowest predicted $Em_{50/50}$. LISMIK **NN** appears frequently in hypothetical complexes with low predicted $Em_{50/50}$. MAXWIS **CN** appears frequently in hypothetical complexes with low predicted lifetime and is present in the hypothetical complex with the third lowest predicted lifetime. MIMYEO **NN** appears frequently in hypothetical complexes with high predicted lifetime. OVALEE **NN** appears frequently in hypothetical

complexes with low predicted lifetime. QEQVOA **NN** appears frequently in hypothetical complexes with low predicted spectral integral. RADTEZ **CN** is present in the three hypothetical complexes with the highest predicted $Em_{50/50}$ and in the hypothetical complex with the second-highest predicted spectral integral. RASGAV **NN** appears frequently in hypothetical complexes with high predicted $Em_{50/50}$ and lifetime and is present in the hypothetical complex with the third-highest predicted spectral integral and the hypothetical complex with the third-highest predicted lifetime. TOTPAW **NN** appears frequently in hypothetical complexes with low predicted $Em_{50/50}$ and lifetime and is present in the hypothetical complex with the highest predicted lifetime and the hypothetical complex with the second-lowest predicted lifetime. TUZHEE **NN** appears frequently in hypothetical complexes with low predicted $Em_{50/50}$ and is present in the hypothetical complex with the second-lowest predicted $Em_{50/50}$. YUWWOD **NN** appears frequently in hypothetical complexes with high predicted spectral integral and $Em_{50/50}$ and is present in the hypothetical complex with the third-highest predicted $Em_{50/50}$ and the hypothetical complex with the second-highest predicted lifetime.

Table S21. The HLS and CSD ligands that appear most often in hypothetical iridium complexes with random split ANN-predicted properties at the high and low extremes, out of the 3,598 hypothetical complexes considered. The six letter identifiers are CSD refcodes. Only complexes within the UQ cutoffs are considered.

		ligand	appearances
$Em_{50/50}$	top 10th percentile	FEQSEB **NN**	31
		YUWWOD **NN**	29
		RASGAV **NN**	25
	bottom 10th percentile	TUZHEE **NN**	27
		LISMIK **NN**	25
		TOTPAW **NN**	23
lifetime	top 10th percentile	RASGAV **NN**	49
		FEQSEB **NN**	43
		MIMYEO **NN**	41
	bottom 10th percentile	TOTPAW **NN**	40
		OVALEE **NN**	37
		MAXWIS **CN**	22
spectral integral	top 10th percentile	YUWWOD **NN**	39
		ACUROI **NN**	35
		**NN33**	29
	bottom 10th percentile	KAFPEO **NN**	54
		**NN37**	27
		QEQVOA **NN**	26

Table S22. The ligands present in the hypothetical complexes with the most extreme random split ANN-predicted properties. The six-letter identifiers are CSD refcodes. 232 out of 3,598 hypothetical complexes have a predicted spectral integral of zero, preventing the identification of just three extreme complexes on the low end of spectral integral. Only complexes within the UQ cutoffs are considered, and for the case of $Em_{50/50}$ and lifetime only complexes that are predicted to be bright (i.e. spectral integral greater than 1×10^5 counts) are considered.

		**CN** ligand	**NN** ligand	predicted value
$Em_{50/50}$	highest	RADTEZ **CN**	**NN43**	2.4669
		RADTEZ **CN**	**NN3**	2.4657
		RADTEZ **CN**	YUWWOD **NN**	2.4647
	lowest	**CN2**	LEZJAD **NN**	1.9682
		**CN101**	TUZHEE **NN**	1.9641
		**CN54**	LEZJAD **NN**	1.9631
lifetime	highest	**CN95**	TOTPAW **NN**	10.4054
		**CN95**	YUWWOD **NN**	10.0016
		**CN67**	RASGAV **NN**	9.8935
	lowest	MAXWIS **CN**	**NN1**	0.0632
		**CN9**	TOTPAW **NN**	0.0517
		**CN101**	CIDDAX **NN**	0.0491
spectral integral	highest	CIGKIP **CN**	**NN24**	2.26E6
		RADTEZ **CN**	**NN20**	2.20E6
		**CN103**	RASGAV **NN**	2.19E6

Table S23. The 26 experimental complexes evaluated with TDDFT in order to benchmark ANN predictions trained on a random split of the data. Here, complexes are represented by the combination of a **CN** and **NN** ligand.

**CN** ligand	**NN** ligand
**CN101**	**NN40**
**CN101**	**NN41**
**CN103**	**NN26**
**CN107**	**NN41**
**CN109**	**NN40**
**CN14**	**NN20**
**CN2**	**NN3**
**CN28**	**NN41**
**CN3**	**NN34**
**CN3**	**NN40**
**CN31**	**NN33**
**CN31**	**NN34**
**CN31**	**NN6**
**CN38**	**NN27**
**CN39**	**NN3**
**CN39**	**NN41**
**CN54**	**NN1**
**CN54**	**NN40**
**CN63**	**NN42**
**CN69**	**NN27**
**CN69**	**NN33**
**CN75**	**NN16**
**CN77**	**NN3**
**CN81**	**NN34**
**CN95**	**NN42**
**CN95**	**NN8**

Table S24. Pearson and Spearman's rank correlation coefficients between experimental properties and random split ANN-predicted properties over 26 representative test set complexes.

	Pearson	Spearman's
$Em_{50/50}$	0.98	0.98
lifetime	0.54	0.82

Table S25. Pearson and Spearman's rank correlation coefficients between experimental properties and B3LYP TDDFT-predicted properties over 26 representative test set complexes.

	geometry	Pearson	Spearman's
$Em_{50/50}$	S0	0.81	0.86
	T1	0.68	0.63
lifetime	S0	0.88	0.89
	T1	0.67	0.44

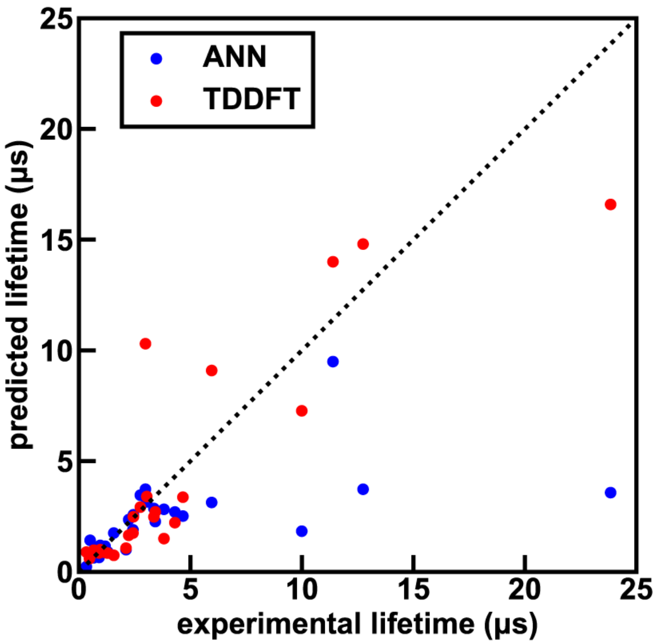

Figure S12. Comparison of random split ANN and TDDFT lifetime prediction to experiment (in μs) across 26 test set iridium complexes in the experimental dataset. These complexes were chosen to span the range of emission energies and lifetimes of the full set. TDDFT was carried out on optimized singlet (S0) geometries using the B3LYP functional. Lifetime was calculated using excitation energy and transition dipole moment output from the TDDFT calculation (see main text *Computational Details*). The dotted line is included as a reference and corresponds to perfect agreement between prediction and experiment.

Table S26. Random split ANN predictions on the bright complexes in the test data with the longest lifetimes.

CN ligand	NN ligand	experimental lifetime (µs)	ANN-predicted lifetime (µs)
**CN101**	**NN40**	23.84	3.58
**CN101**	**NN41**	12.74	3.73
**CN95**	**NN37**	12.38	7.59
**CN95**	**NN8**	11.39	9.49
**CN95**	**NN3**	11.23	10.26
**CN28**	**NN40**	10.9	2.17
**CN28**	**NN41**	9.99	1.83
**CN95**	**NN42**	5.95	3.13
**CN11**	**NN20**	5.2	3.93
**CN34**	**NN34**	5.11	6.30
**CN38**	**NN27**	4.66	2.52
**CN29**	**NN33**	4.58	2.87
**CN42**	**NN40**	4.5	2.70
**CN35**	**NN20**	4.38	2.72
**CN38**	**NN26**	4.33	3.49

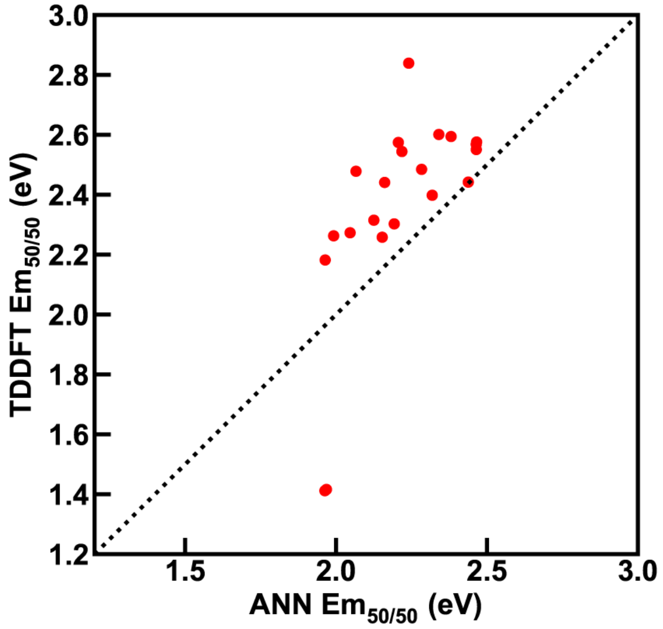

Figure S13. Comparison of random split ANN and TDDFT $Em_{50/50}$ predictions (in eV) across 21 hypothetical iridium complexes. TDDFT was carried out on optimized singlet (S0) geometries using the B3LYP functional. The energy of the three lowest triplet sublevels was averaged for the TDDFT energy, which is used to approximate $Em_{50/50}$.

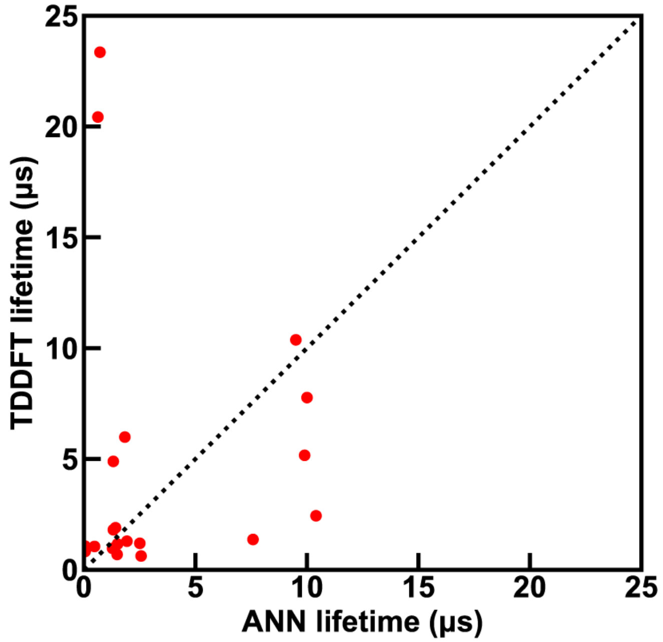

Figure S14. Comparison of random split ANN and TDDFT lifetime predictions (in µs) across 21 hypothetical iridium complexes. TDDFT was carried out on optimized singlet (S0) geometries using the B3LYP functional. Lifetime was calculated using excitation energy and transition dipole moment output from the TDDFT calculation (see main text *Computational Details*).

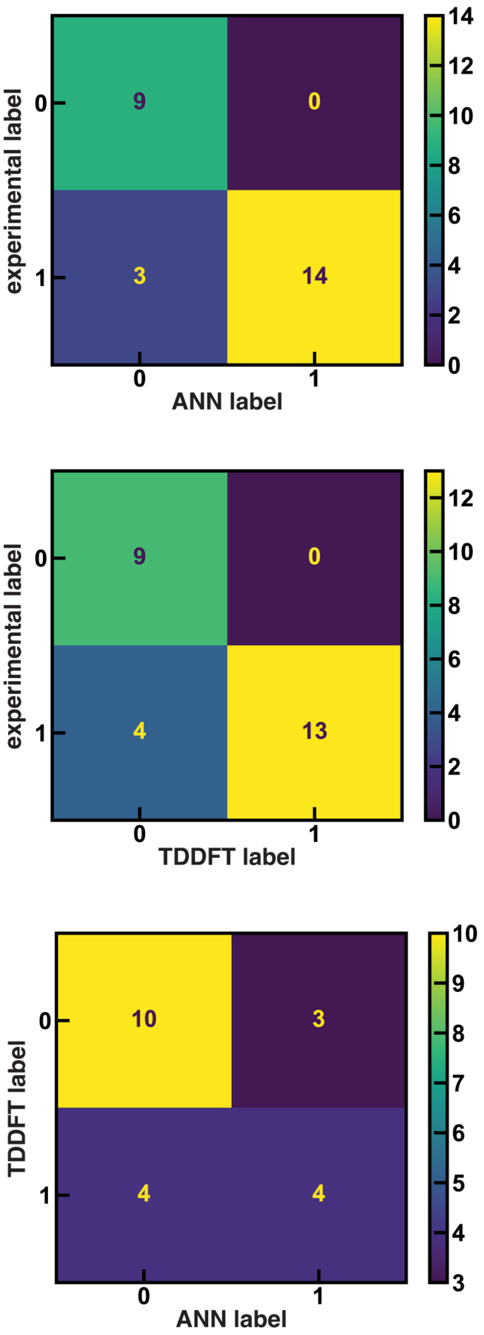

Figure S15. Confusion matrices indicating the agreement between different lifetime labels. 0 corresponds to a lifetime $\tau <= 2$ μs and 1 corresponds to $\tau > 2$ μs. Over the 26 representative test set experimental complexes, we show experimentally measured lifetimes versus random split ANN predictions (top) and TDDFT predictions (middle). Over the 21 hypothetical complexes, we show TDDFT predictions versus random split ANN predictions (bottom). The TDDFT results were generated with singlet (S0) geometries and the B3LYP functional.

Table S27. The 21 hypothetical complexes evaluated with TDDFT in order to benchmark random split ANN predictions. Here, complexes are represented by the combination of a **CN** and **NN** ligand.

**CN** ligand	**NN** ligand
**CN101**	CIDDAX_eq_lig_0
**CN101**	TUZHEE_eq_lig_2
**CN107**	MUTMOF_eq_lig_2
**CN2**	LEZJAD_eq_lig_2
**CN54**	LEZJAD_eq_lig_2
**CN67**	RASGAV_eq_lig_2
**CN67**	TOTPAW_eq_lig_0
**CN76**	REWDII_eq_lig_0
**CN79**	GEMXAZ_eq_lig_2
**CN9**	TOTPAW_eq_lig_0
**CN95**	MIMYEO_eq_lig_0
**CN95**	TOTPAW_eq_lig_0
**CN95**	YUWWOD_eq_lig_2
HALLEO_ax_lig_0	FEQSEB_eq_lig_2
MAXWIS_ax_lig_0	**NN1**
OJUSET_ax_lig_0	TUZHEE_eq_lig_2
RADTEZ_ax_lig_0	**NN3**
RADTEZ_ax_lig_0	**NN33**
RADTEZ_ax_lig_0	YUWWOD_eq_lig_2
RANGOE_ax_lig_0	**NN47**
SUHLOZ_ax_lig_0	**NN34**

Table S28. Pearson and Spearman's rank correlation coefficients between random split ANN-predicted properties and B3LYP TDDFT-predicted properties. Type indicates whether the correlation coefficients are evaluated over the 26 representative phosphors from the experimental dataset or over the 21 representative hypothetical phosphors.

	geometry	type	Pearson	Spearman's
$Em_{50/50}$	S0	experimental	0.81	0.89
	S0	hypothetical	0.67	0.79
	T1	experimental	0.67	0.65
	T1	hypothetical	0.65	0.75
lifetime	S0	experimental	0.71	0.85
	S0	hypothetical	0.05	0.32
	T1	experimental	0.79	0.54
	T1	hypothetical	-0.21	-0.13

Table S29. The MAE, scaled MAE, and R^2 test set performance of eight random split ANNs predicting for $Em_{50/50}$, where a 70/30 train/test split is used instead of the 85/15 split from Table S11.

	MAE (eV)	scaled MAE	R^2
Morgan	0.0159	0.031	0.93
Dice	0.0165	0.032	0.93
RAC	0.0300	0.057	0.82
ligand-only RAC	0.0222	0.043	0.91
CD-RAC	0.0255	0.049	0.86
xTB	0.0258	0.050	0.88
B3LYP DFT	0.0337	0.065	0.80
ωPBEh DFT	0.0274	0.053	0.86

Table S30. The most different HLS ligands as measured by Dice similarities of Morgan fingerprints. For each **CN** (**NN**) ligand in the HLS, its similarity with each other **CN** (**NN**) ligand in the HLS was taken. For each ligand, these similarities were then averaged to yield a metric for the similarity of that ligand to the rest of the HLS. The lowest averages of similarities are reported below. The three most different **CN** ligands and the two most different **NN** ligands were used to form an out-of-distribution grouped split test set.

**CN** ligand	average Dice similarity	**NN** ligand	average Dice similarity
CN103	0.274	***NN43***	0.210
CN104	0.290	***NN20***	0.251
CN21	0.300	**NN21**	0.274
**CN108**	0.305	**NN24**	0.277
**CN109**	0.312	**NN34**	0.282

Table S31. Hyperopt-selected hyperparameters for the best-performing random split ANNs. The lists in the architecture row indicate the number of hidden layers and the number of nodes in each layer. Learning rate, beta1, and decay all affect the Adam optimizer that we use when training our ANNs. The label "bypass" indicates the presence of layers that concatenate inputs from the input layer and non-adjacent ANN layers. res indicates the presence of layers that add inputs from non-adjacent ANN layers.

	Dice feature set			xTB feature set		
	$Em_{50/50}$	lifetime	spectral integral	$Em_{50/50}$	lifetime	spectral integral
architecture	(256, 256, 256)	(512, 512, 512)	(512, 512, 512)	(512, 512, 512)	(256, 256, 256)	(256, 256, 256)
learning rate	0.00069	0.00089	0.00020	0.00085	0.00080	0.00068
beta1	0.880	0.811	0.982	0.827	0.848	0.873
decay	0.00179	0.00321	0.00039	0.00124	0.00035	0.00246
L2 regularization	0.0105	0.0051	0.0333	0.0044	0.0041	0.0654
dropout rate	0.354	0.356	0.256	0.203	0.294	0.054
batch size	32	16	16	256	128	256
epochs	1122	1052	1085	2000	1152	2000
bypass	True	False	True	True	True	True
res	True	True	True	True	False	False

Table S32. Statistics on structure attrition for hit CSD complexes used to identify **CN** and **NN** ligands outside of the HLS. There were six reasons why any CSD complex could be eliminated from consideration: the presence of multiple iridium atoms; the absence of any iridium atoms due to the presence of another molecule larger than the iridium complex in the CSD entry (combined with the “Export largest molecule only” option, as described in Text S1); the incomplete removal of solvent or counterions leading to index errors; the presence of non-bidentate ligands such that a complex was not 2-2-2, i.e. did not have three bidentate ligands in octahedral geometry; refcode duplicates such as HULVEQ and HULVEQ01; and the presence of a **CC** ligand.

starting complexes	multiple Ir atoms	no Ir atoms	solvent/ counterion	not 2-2-2	refcode duplicates	presence of a **CC** ligand	final complexes
700	33	4	7	32	5	1	618

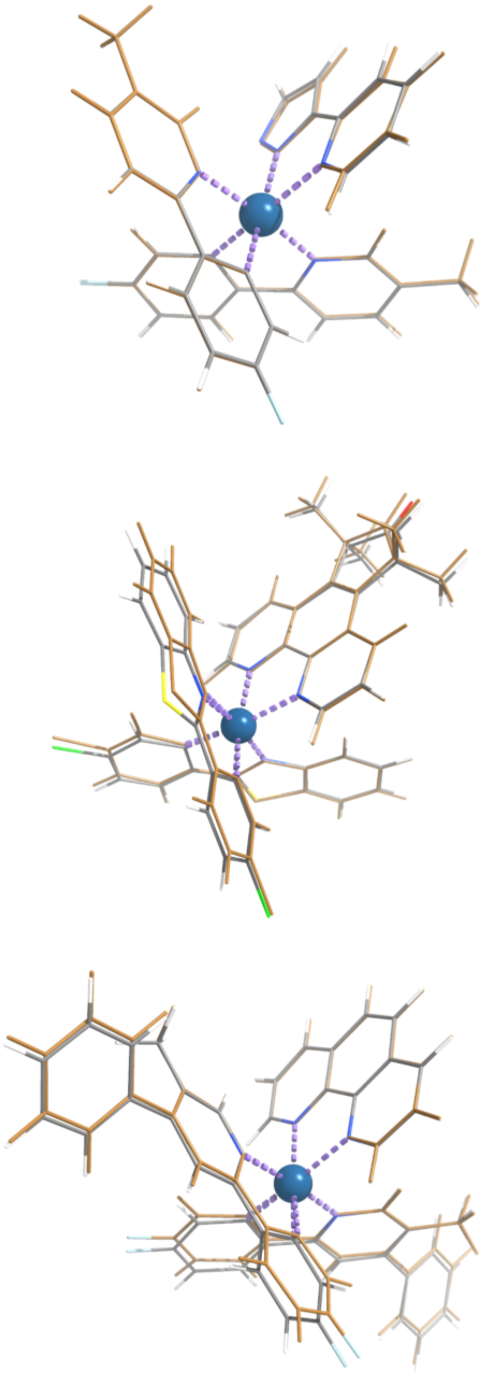

Figure S16. Comparison of singlet (S0) and triplet (T1) B3LYP geometries of select iridium complexes, [Ir(**CN3**)$_2$(**NN40**)]^0 (top), [Ir(**CN38**)$_2$(**NN27**)]$^+$ (middle), and [Ir(**CN75**)$_2$(**NN16**)]$^+$ (bottom). Triplet geometries are shown in brown, while singlet geometries are colored normally with white for hydrogen, gray for carbon, blue for nitrogen, red for oxygen, light blue for fluorine, yellow for sulfur, green for chlorine, and dark blue for iridium. The RMSD values between these singlet-triplet structure pairs are 0.065 Angstroms, 0.188 Angstroms, and 0.164 Angstroms, respectively.

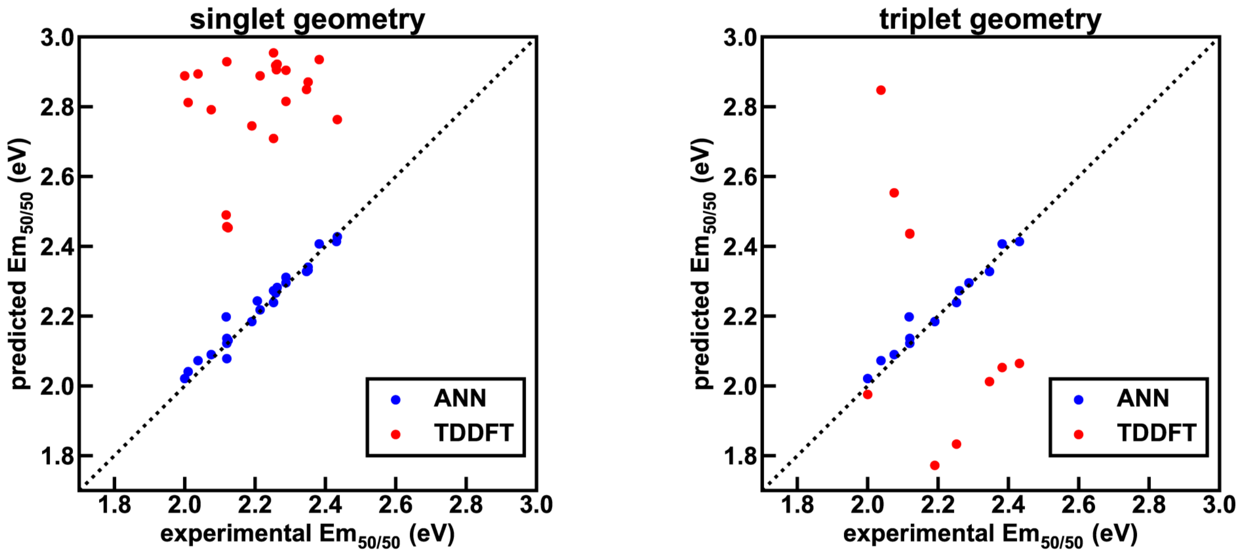

Figure S17. TDDFT emission energy predictions with the CAM-B3LYP functional, on iridium phosphor geometries optimized with the CAM-B3LYP functional in the singlet (left; S0) and triplet (right; T1) state.

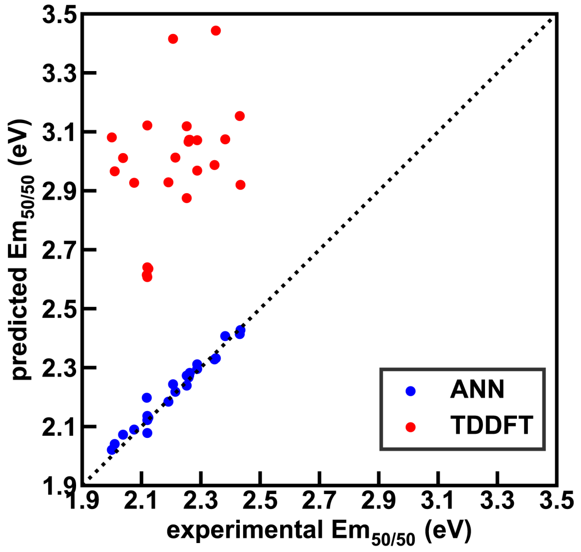

Figure S18. TDDFT emission energy predictions with the ωB97X-D3BJ functional, on iridium phosphor geometries optimized with the ωB97X-D3BJ functional in the singlet (S0) state.

References

(1) DiLuzio, S.; Mdluli, V.; Connell, T. U.; Lewis, J.; VanBenschoten, V.; Bernhard, S. High-Throughput Screening and Automated Data-Driven Analysis of the Triplet Photophysical Properties of Structurally Diverse, Heteroleptic Iridium(III) Complexes. *J. Am. Chem. Soc.* **2021,** *143*, 1179-1194.

(2) Grimme, S.; Bannwarth, C.; Shushkov, P. A Robust and Accurate Tight-Binding Quantum Chemical Method for Structures, Vibrational Frequencies, and Noncovalent Interactions of Large Molecular Systems Parametrized for All spd-Block Elements (Z= 1–86). *J. Chem. Theory Comput.* **2017,** *13*, 1989-2009.

(3) Ásgeirsson, V.; Bauer, C. A.; Grimme, S. Quantum Chemical Calculation of Electron Ionization Mass Spectra for General Organic and Inorganic Molecules. *Chem. Sci.* **2017,** *8*, 4879-4895.

(4) Janet, J. P.; Kulik, H. J. Resolving Transition Metal Chemical Space: Feature Selection for Machine Learning and Structure–Property Relationships. *J. Phys. Chem. A* **2017,** *121*, 8939-8954.

(5) Harper, D. R.; Nandy, A.; Arunachalam, N.; Duan, C.; Janet, J. P.; Kulik, H. J. Representations and Strategies for Transferable Machine Learning Improve Model Performance in Chemical Discovery. *Journal of Chemical Physics* **2022,** *156*, 074101.

(6) Rogers, D.; Hahn, M. Extended-Connectivity Fingerprints. *J. Chem. Inf. Model.* **2010,** *50*, 742-754.

(7) Cereto-Massagué, A.; Ojeda, M. J.; Valls, C.; Mulero, M.; Garcia-Vallvé, S.; Pujadas, G. Molecular Fingerprint Similarity Search in Virtual Screening. *Methods* **2015,** *71*, 58-63.

(8) Duan, C.; Liu, F.; Nandy, A.; Kulik, H. J. Data-Driven Approaches Can Overcome the Cost–Accuracy Trade-Off in Multireference Diagnostics. *J. Chem. Theory Comput.* **2020,** *16*, 4373-4387.

(9) You, Y.; Park, S. Y. Phosphorescent Iridium(III) Complexes: Toward High Phosphorescence Quantum Efficiency through Ligand Control. *Dalton Trans.* **2009**, 1267-1282.

(10) Landrum, G. RDKit Documentation. *Release* **2013,** *1*, 4.

(11) Janet, J. P.; Duan, C.; Yang, T.; Nandy, A.; Kulik, H. J. A Quantitative Uncertainty Metric Controls Error in Neural Network-Driven Chemical Discovery. *Chem. Sci.* **2019,** *10*, 7913-7922.
